# Proof of concept nanotechnological approach to in vitro targeting of malignant melanoma for enhanced immune checkpoint inhibition

**DOI:** 10.1038/s41598-023-34638-2

**Published:** 2023-05-08

**Authors:** Bandar Alharbi, Husam Qanash, Naif K. Binsaleh, Salem Alharthi, Abdulbaset M. Elasbali, Chandranil H. Gharekhan, Muhammad Mahmoud, Emmanouil Lioudakis, John J. O’Leary, Derek G. Doherty, Bashir M. Mohamed, Steven G. Gray

**Affiliations:** 1 Department of Medical Laboratory Science, College of Applied Medical Sciences, University of Ha’il, Hail, 55476 Saudi Arabia; 2 Department of Biological Science, College of Arts and Science, Najran University, Najran, 55461 Saudi Arabia; 3 Clinical Laboratory Science, College of Applied Medical Sciences-Qurayyat, Jouf University, Sakaka, 42421 Saudi Arabia; 4 Amrita Center for Nanosciences and Molecular Medicine, Amrita Vishwa Vidyapeetham, Cochin, India; 5 School of Medicine, Trinity College Dublin, Dublin, Ireland; 6 Department of Pharmacology and Therapeutics, School of Medicine, Trinity College Dublin, Dublin, Ireland; 7 Department of Histopathology, Trinity College Dublin, Emer Casey Molecular Pathology Research Laboratory, Coombe Women and Infants University Hospital, Dublin, Ireland; 8 Trinity St James’s Cancer Institute, Dublin, Ireland; 9 Department of Obstetrics and Gynaecology, Trinity College Dublin, Dublin, Ireland; 10 Department of Immunology, Trinity College Dublin, Dublin, Ireland; 11 Department of Clinical Medicine, Trinity College Dublin, Dublin, Ireland

**Keywords:** Cancer, Immunology, Nanoscience and technology

## Abstract

Immunotherapies, including immune checkpoint inhibitors, have limitations in their effective treatment of malignancies. The immunosuppressive environment associated with the tumor microenvironment may prevent the achievement of optimal outcomes for immune checkpoint inhibitors alone, and nanotechnology-based platforms for delivery of immunotherapeutic agents are increasingly being investigated for their potential to improve the efficacy of immune checkpoint blockade therapy. In this manuscript, nanoparticles were designed with appropriate size and surface characteristics to enhance their retention of payload so that they can transmit their loaded drugs to the tumor. We aimed to enhance immune cell stimulation by a small molecule inhibitor of PD-1/PD-L1 (BMS202) using nanodiamonds (ND). Melanoma cells with different disease stages were exposed to bare NDs, BMS202-NDs or BMS202 alone for 6 h. Following this, melanoma cells were co-cultured with freshly isolated human peripheral blood mononuclear cells (hPBMCs). The effects of this treatment combination on melanoma cells were examined on several biological parameters including cell viability, cell membrane damage, lysosomal mass/pH changes and expression of γHA2X, and caspase 3. Exposing melanoma cells to BMS202-NDs led to a stronger than normal interaction between the hPBMCs and the melanoma cells, with significant anti-proliferative effects. We therefore conclude that melanoma therapy has the potential to be enhanced by non-classical T-cell Immune responses via immune checkpoint inhibitors delivered by nanodiamonds-based nanoparticles.

## Introduction

In 2020 the number of new cases of melanoma worldwide was estimated to be 325,000^1^, and if rates continue to increase by 2040, 510,000 new cases and 96,000 deaths are estimated^1^. The five-year survival rate is about 90%, with the prognosis being best for patients who are diagnosed at an early stage without any metastatic disease^2^. A complementary approach to improve the survival rate of patients with metastatic melanoma is the usage of immune-stimulating monoclonal antibodies, which suppress endogenous inhibitors of the immune response: ipilimumab that blocks CTLA-4^3,4^ and nivolumab that blocks the PD-1 receptor^5–9^.

Programmed death-1 (PD-1) is expressed on the surface of T cells, B cells, NK cells, monocytes, and dendritic cells^10,11^. The interaction between PD-1 and its ligand PD-L1 plays a vital role in preserving self-tolerance and thereby avoiding autoimmunity, but this interaction can prevent immune-mediated rejection of tumor cells. PD-1/PD-L1 blockade has been revolutionary in cancer immunotherapy, and it has been used in the treatment of numerous malignancies, including melanoma^12^, hepatocellular carcinoma^13^, non-small cell lung cancer^14,15^, breast cancer^16^, as well as Hodgkin’s lymphoma^17^. However, only a minority of patients (20–30%) are estimated to show a positive response to PD-1/PD-L1 blockade therapy^18^. Patients may also acquire resistance which could eventually lead to cancer progression in patients who have had a clinical response^19–21^. Therefore, there remains an unmet need to improve the present current treatments.

Currently, all the approved immune checkpoint inhibitors (ICI) are based on monoclonal antibodies (mABs), as incomplete structural information impeded small molecule inhibitor development^22^. However, small molecule inhibitors are emerging, and the best characterized of these is a compound developed by Bristol Myers Squibbs (BMS-202), which is a potent small molecule that causes PD-L1 receptor dimerization, preventing PD-1 interactions^23^. This has led to the rationale design of other small molecule inhibitors targeting the PD-L1 dimer^24–27^. The potential efficacy of BMS-202 as a therapy in melanoma has been investigated using a syngeneic mouse melanoma model^28^, although it must be noted that direct off-target cytotoxic effects of BMS-202 have also been recorded in other cancer cell line experiments^29^.

**Figure 1:**
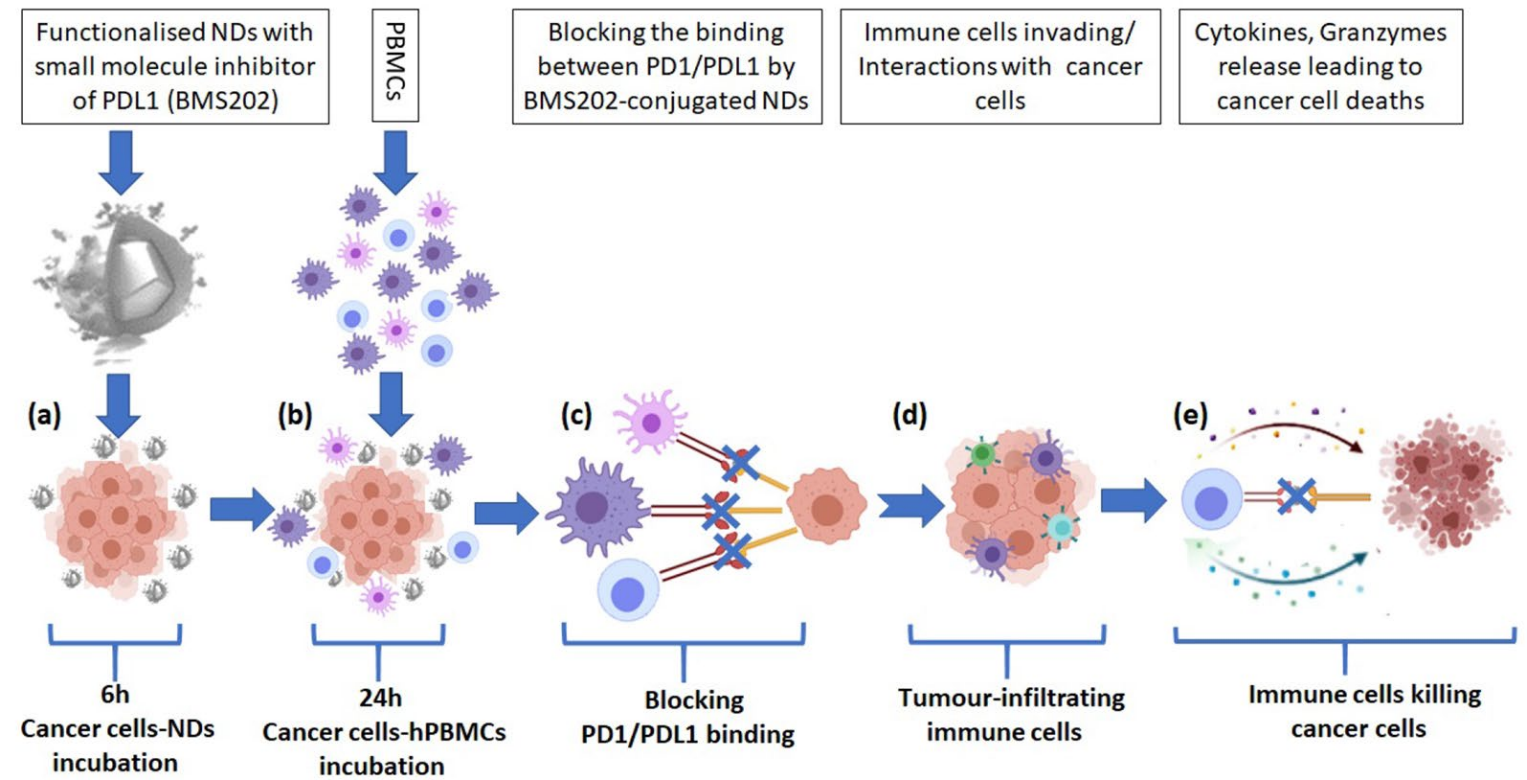
Schematic diagram illustrating the enhancement of immune cells infiltration and their anticancer activities. (**a**) Exposure to BMS202-conjugated NDs for 6 h. (**b** and **d**) Melanoma cells co-cultured with hPBMCs for an additional 24 h. (**c**) Immune cells/cancer cells interaction. (**e**) Induction of cytotoxicity by Immune cells.

Nanomaterials are under investigation as a means to achieve targeted drug delivery to treat cancers^30,31^. The main advantage of delivering drugs via nanoparticle delivery systems is the high efficacy of the treatment coupled with less side effects as the therapy is delivered specifically to where it is required, and more recently is being actively pursued as a means to improve immune checkpoint blockade efficiency^32^. The rationale for the current study is based on several reports examining the potential for using nanotechnology to both improve drug delivery and enhance immune checkpoint responses in cancer models including melanoma^26,27,33–37^. Nanodiamonds are considered to be a useful nanomaterial for the development of such drug delivery platforms on the basis of their stability, clearance and non-toxicity^38–43^. A proof-of-concept drug delivery system was developed based on nanodiamonds loaded with a small molecule inhibitor of PD-1/PD-L1 immune checkpoint inhibitor (ICI) (BMS202)^23,29,44^ (Fig. 1).

Subsequently we evaluated their ability to elicit enhanced cell-mediated anticancer responses through immune cell responses in vitro, using melanoma cell lines, co-cultured with non-HLA typed human peripheral blood mononuclear cells (hPBMCs).

## Materials and methods

### Ethics

The research study/protocol was approved by St. James’s Hospital, and Adelaide and Meath Hospital, Dublin, incorporating the National Children’s Hospital Research Ethics (SJH/AMNCH) Committee Dublin (20,170,908). Peripheral blood mononuclear cells were prepared from anonymized healthy human buffy coat packs obtained from the Irish Blood Transfusion Service (IBTS, St. James’s Hospital, Dublin, Ireland). The experiments were performed in accordance with the Helsinki declaration and relevant guidelines and regulations. Informed consent was obtained from all subjects and/or their legal guardians. All methods in this study were performed in accordance with the relevant guidelines and regulations.

### Nanocomplex development

BMS202 (Cat. No. S7912-SEL—10 mM in 1 ml of DMSO) was purchased from Stratech (Stratech, Ely, United Kingdom). 100 μg of Nanodiamond (ND) powder (provided as a gift from Nanodiamond Products Limited, Ireland, (now Hyperion Materials and Technologies) were re-suspended in deionized water (DW) to form NDs solution; then, this suspension was autoclaved. The MSDS and associated safety profiles for these NDs can be obtained by contacting the company directly at the following link: https://www.hyperionmt.com/contact/sds/.

Following this, purified NDs were PEGylated using 0.2 mM 2-methoxy (poly-ethyleneoxy)-propyl trimethoxy saline (Cat. No. S2535, Cymit Química S. L. Barcelona, Spain).

As previously described^45^, PEGylated NDs were mixed with 50 mM 2-(N-morpholino) ethanesulfonic acid (1.0 M MES, Cat. No. J60763.AK, ThermoFisher Scientific, Dublin, Ireland) buffer (pH 6) for 24 h. ND-PEG were then concentrated and re-suspended in 0.5 mL of MES buffer containing 10.0 mg of N-Ethyl-N'-[3-dimethylaminopropyl] carbodiimide (EDC—Cat. No. PG82079—ThermoFisher Scientific, Dublin, Ireland) and 10.0 mg of N-hydroxysuccinimide (NHS, Cat No. 24500—ThermoFisher Scientific, Dublin, Ireland) and vigorously agitated for 15 min. Then, the reaction mixture was washed twice by centrifugation, at 13,300 rpm, with deionized water (DW) and re-suspended in 1 mL MES buffer containing 100ul of (10 mM BMS202). The reaction mixture was agitated for 4 h at RT. The particles were then washed with DW, re-suspended in purified DW producing the nanocarrier complex termed ND/BMS202 (Fig. 1). Following that, ND and ND-PEG-BMS202 were physicochemically characterized by a dynamic light scattering (DLS) detector (Zetasizer Nano ZS90, Malvern).

### Isolation of hPBMCs

Human peripheral blood mononuclear cells (hPBMCs) were isolated from the peripheral blood of a single individual by density gradient separation through Ficoll-Paque Plus (Cat. No. 17–1440-02 Cytiva) according to the manufacturer’s instructions. hPBMCs were cultured for 24 h in complete medium composed of RPMI 1640 containing human fetal calf serum (10%), glutamine (1%), penicillin and streptomycin (1%) (Sigma-Aldrich, Ireland) prior to use in experiments. All experiments were carried out in triplicate.

### Cancer cell culture and reactivity assay of hPBMCs

Three isogenic melanoma cell lines showed similar patterns: WM793 (poorly tumorigenic parental cells), WM793P1 (more tumorigenic derivative) and 1205Lu (metastatic derivative)^46^ were seeded at a density of 5000 cells per well in 96 well plates overnight and subsequently treated as follows.

- Melanoma cells exposed to nanocomplexes and hPBMCs.
- Melanoma cells exposed to BMS202 and hPBMCs.
- Melanoma cells exposed solely to hPBMCs.
- Melanoma cells exposed solely to nanocomplexes (BMS202-NDs).
- Melanoma cells exposed solely to BMS202.

Treatments involved exposure for 6 h to our lab-developed nanocomplexes (BMS202-ND) at concentrations of 2.5 µM, 5 µM, 10 µM and to BMS202 alone at a concentration of 10 µM. Unexposed cells and cells exposed to bare NDs were used as controls. Following this, 50,000 hPBMCs were added per well (at a ratio of 10 hPBMCs/1 cancer cell). After treatment, cells were repeatedly washed with phosphate buffered saline (PBS) and fixed with 3% paraformaldehyde (PFA) for 20 min at room temperature followed by washing in PBS two times for 5 min each. They were then stained for actin and an immune cell marker such as CD8^+^ T cells and CD45 and imaged using an inverted microscope.

### Cell viability and proliferation changes

Following exposure of melanoma cell lines to either bare NDs, NDs/hPBMCs, BMS202-NDs/hPBMCs or BMS202/hPBMCs, the cells were fixed as above and counterstained with Hoechst 33,342 (HQ) for visualization of cell nuclei (Cat. No. 62249 HQ; 1:1000 dilution; ThermoFisher Scientific, Dublin, Ireland) by incubation for 20 min at room temperature (RT). To quantify the number of cells, untreated controls (NT) and treated cells with BMS202-NDs/hPBMCs were imaged using an inverted fluorescent microscope and the resulting images were scanned and analyzed using the Cytell™ imaging system (GE Healthcare, UK) as previously described^47^.

### Cell membrane permeability and lysosomal mass/pH changes

It is well known that some toxic agents can induce damage to the cell membrane and inhibit the cell’s functionality by affecting the pH of organelles such as lysosomes and endosomes, or by causing an increase in the number of lysosomes^48–50^. Following exposure to bare NDs, NDs/hPBMCs, BMS202-NDs/hPBMCs and BMS202/hPBMCs, cells were imaged using an inverted fluorescent microscope and changes in cell membrane permeability and lysosomal mass/pH scanned and analyzed using the Cytell™ imaging system. An example figure and description of the methodology used for Cytell™ analysis is provided in Supplementary Fig. S1.

### DNA damage

Treated melanoma cells were washed with PBS and fixed with 3% PFA for 20 min at room temperature (RT) then washed with PBS. Cells were blocked with 2% BSA/0.1% Triton-X in PBS for 20 min at RT, and then probed with rabbit anti-γ-H2AX antibodies (Cat. 9718, Cell Signaling Technology, Ireland), diluted 1:200 in 0.5% BSA/0.1% Triton-X/PBS then incubated for 24 h at 4 °C. Cells were washed with PBS and incubated with goat anti-rabbit antibody (Cat. No. A32731, Thermofisher Scientific, Dublin, Ireland) for 1 h at RT. Following this, cells were washed with PBS and counterstained with Hoechst 33,342 for visualization of cell nuclei (Cat. No. 62249; HQ; 1:1000 dilution; ThermoFisher Scientific, Dublin, Ireland) by incubation for 20 min at RT. To quantify the percentage of cells expressing γ-H2AX, controls and treated cells with BMS202-ND were scanned and analyzed using the Cytell™ imaging system.

### Immunoblotting

Melanoma cells were cultured in T-25 flasks and exposed to 2.5 µM, 5 µM, 10 µM of BMS202-NDs or to 10 µM of BMS202 alone for 6 h and then co-cultured with 100,000 hPBMCs for an additional 24 h as described above. Cell lysates were extracted in RIPA buffer supplemented with a protease inhibitor cocktail (Cat. No. P8340-1ML Sigma-Aldrich, Ireland). The resulting lysates were centrifuged at 16,000 × g for 20 min at 4 °C and the protein concentration of the supernatants was determined by a Bradford assay. 30ug of cell lysates from each treated sample was resolved by sodium dodecyl sulphate polyacrylamide gel electrophoresis (SDS-PAGE). Then samples were transferred to a nitrocellulose membrane and blocked with LI-COR blocking buffer (Cat. No. 927–60,001, LI-COR Biosciences UK Ltd) for 1 h at 4 °C. The membranes were then probed with rabbit anti-cleaved caspase 3 (Cat. 96,645, Cell Signaling Technology, Ireland, and anti-γ-H2AX antibodies (Cat. 9718, Cell Signaling Technology, Ireland), at a concentration of 1 in 500 in Intercept^®^ T20 (TBS) antibody diluent (Cat. No. 927–85,001, LI-COR Biosciences UK Ltd) overnight at 4 °C. After washing with TBST, membranes were incubated with secondary antibodies conjugated to a fluorescent entity: IRDye 800-conjugated goat anti-rabbit IgG (1:1000—Cat. No. 926–32,211, LI-COR Biosciences UK Ltd) and IRDye-680-conjugated goat anti-mouse IgG (1:2000) in blocking buffer, (Cat. No. 926–68,070, LI-COR Biosciences UK Ltd) for 1 h at 4 °C. For loading control, membranes were washed and re‐probed with GAPDH rabbit monoclonal antibody (Cat. No. 926–42,216, LI-COR Biosciences UK Ltd) An EZ‐RUN™ pre‐stained molecular weight ladder (Cat. No. BP3603 Fisher Scientific, Dublin, Ireland) was used for molecular weight determination. The protein bands were visualized and analyzed on the Odyssey IR imaging system (LI-COR Biosciences) with the image analyst blinded with respect to group designation, and results normalized to GAPDH expression.

### Immunofluorescent microscopy

Melanoma cells were cultured in 96 well plates and then exposed to 2.5 µM, 5 µM, 10 µM of BMS202-NDs or to 10 µM of BMS202 alone for 6 h and then co-cultured with 100,000 hPBMCs for an additional 24 h. Then hPBMCs/melanoma cell lines were washed, fixed in 3% paraformaldehyde (PFA), and then stained with cytotoxic T cell marker (anti-CD8 antibodies), whereas melanoma cell lines were probed with cell tracker (green—C7025, CMFDA Dye, ThermoFisher Scientific, Dublin, Ireland).

### Statistical analysis

All the raw data from the investigated biological parameters were analyzed using GraphPad Prism 8. Statistical significance was determined using one-way ANOVA coupled with a non-parametric Kruskal–Wallis test. For all such analyses nanodiamonds treatment were set as the comparator to which all other treatments were compared for statistical significance. For cell cycle analysis, 2-way ANOVA was carried out. The results are expressed as the mean ± standard error of the mean (n = 3). A value of *p* < 0.05 was considered statistically significant. The resulting p values were reported using the following symbols: * = *p* < 0.05, ** = *p* < 0.01, and *** = *p* < 0.001.

## Results

### *Characterization of* BMS202-ND

The size distribution of our developed nanocomplexes was also determined by DLS. As illustrated in Table 1, in comparison to bare ND (82.0 nm), PEGylated-ND conjugated with-BMS202 (ND-PEG-BMS202) showed an increase in the average size distribution (209.3 nm) with a zeta potential of − 11.8 ± 2.2 mV, which demonstrated that ND-PEG-BMS202 nanocomplexes had higher zeta potentials than bare ND (− 34.2 ± 4.1) in deionized water. Measurements with DLS system indicated that these nanodiamonds had polydispersity indices (PDI) of 0.151 for bare ND and 0.119 for ND-PEG-BMS202 nanocomplexes. The developed nanocomplexes remained in solution for more than 6 months with no apparent aggregation or precipitation of prepared complex, indicating the stability of BMS202-ND (Supplementary Fig. S2).

**Table 1:** Physical–chemical characterization and hydrodynamic size distribution of bare NDs and BMS 202-loaded NDs.

	Diameter (nm)	Zeta potentials (mV)	Polydispersity indices (PDI)
ND	82.0 ± 3.5	− 34.2 ± 4.1	0.151
BMS202-ND	209.3 ± 5 2	− 11.8 ± 2.2	0.119

### PBMCs/melanoma cells interactions

**Figure 2:**
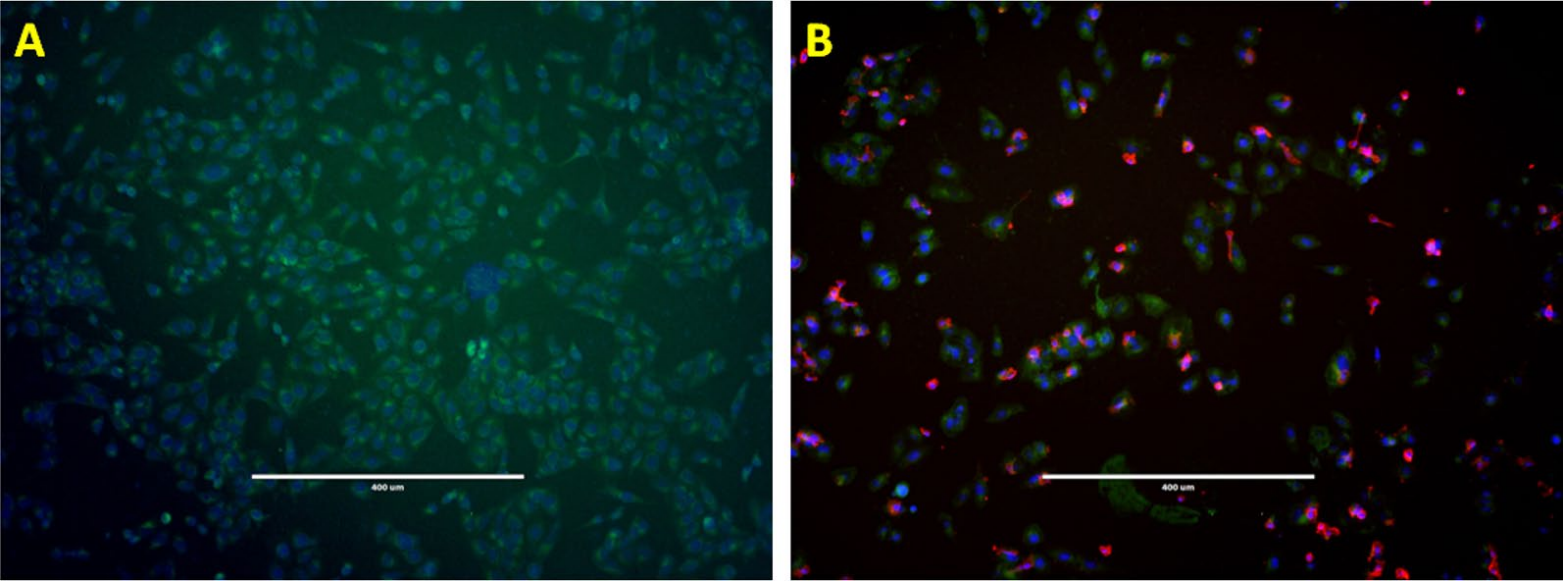
Representative images of hPBMCs/CD+ T cells and Melanoma cells interactions. WM793P1 Melanoma cells were exposed to BMS202-NDs for 6 h and then incubated with hPBMCs for an additional 24 h. (**A**) Non-ND treated cells, (**B**) ND-treated and hPBMCs co-cultured cells were stained with anti-CD8 antibodies (red). Cells then were labelled with cell tracker (green, CMFDA Dye, ThermoFisher Scientific, Dublin, Ireland) and counterstained with Hoechst 33,342, (blue) for visualization of cell nuclei, and indicating interaction of Immune cells with cancer cells. Imaging was performed using an inverted microscope (10X), and scale bar represents 400 µm.

Following treatments, to quantify the level of immune cell activity, we examined the viability of cells exposed to nanodiamond, nanodiamond complexes or drug alone. As illustrated in Fig. 2A significant number of hPBMCs interacted with these melanoma cell lines post-exposure to BMS202-NDs. These hPBMCs were stained with a marker for cytotoxic T cells and natural killer cells (anti-CD8 antibody) the interacting hPBMCs and were found to comprise mainly CD8^+^ T cells (Fig. 2A,B; Supplementary Fig. S3).

### Cell viability and proliferation assay post exposure to ND/BMS202/PBMCs and interaction with hPBMCs

**Figure 3:**
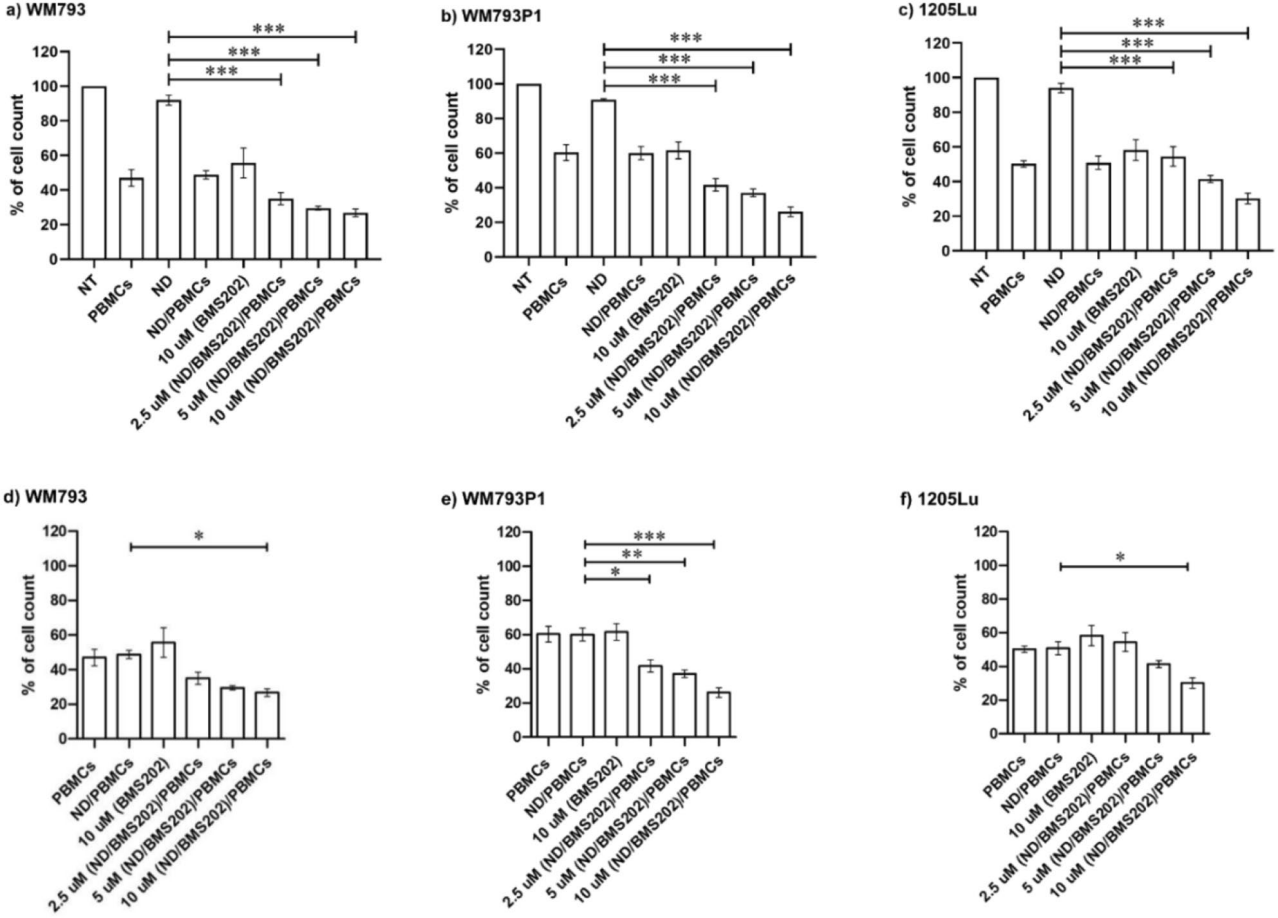
Cell viability inhibition post-exposure to and interaction with hPBMCs. Melanoma cell lines were either left untreated (NT) or treated with either nanodiamonds (NDs) alone; BMS202 alone (10 µM), or ND-BMS202 (2.5 µM, 5 µM, 10 µM) for 6 h, then subsequently co-cultured with hPBMCs for an additional 24 h. Cells were stained with Hoechst 33,342 and scanned and analyzed using the Cytell™ imaging system. Viable cells were automatically counted, and data were presented as mean ± SEM (n = 3). (**a**–**c**) Overall effects on overall cell viability for all treatments. (**d**–**f**) comparison of nanocomplexes compared to ND/PBMCs exposure and continue to show significantly enhanced effects on cell viability. All data were analyzed using one‐way ANOVA with Tukey’s post-hoc test, “**” for *p* < 0.01.

Significant changes of cell viability were seen when melanoma cells were co-cultured with hPBMCs alone and when melanoma cells were co-cultured with BMS202-ND nanocomplexes alone, whereas no changes were observed when cells were exposed to BMS202 alone or NDs alone in comparison to untreated control (Fig. 3a–f). However, when pre-exposed to ND-BMS202 and then co-cultured with hPBMCs a greater decrease was observed for cell viability compared to hPBMCs alone, which was found to be in a concentration-dependent manner (at 2.5 µM; *p* < 0.03, at 5 µM; *p* < 0.005, at 10 µM; *p* < 0.005, respectively) and a cell type-dependent manner (Supplementary Table 1).

When reanalyzed by comparing hPBMCs against ND-BMS202 plus hPBMCs, significant changes to cell proliferation remained in many instances (Supplementary Table 2), confirming that when complexed as a nanoparticle additional anticancer efficacy for the PD-1/PD-L1 small molecule inhibitor BMS202 is achieved.

### Induction of cell membrane permeability damage upon exposure of melanoma cells to bare NDs, NDs/hPBMCs, nanocomplexes/hPBMCs and BMS202/hPBMCs

**Figure 4:**
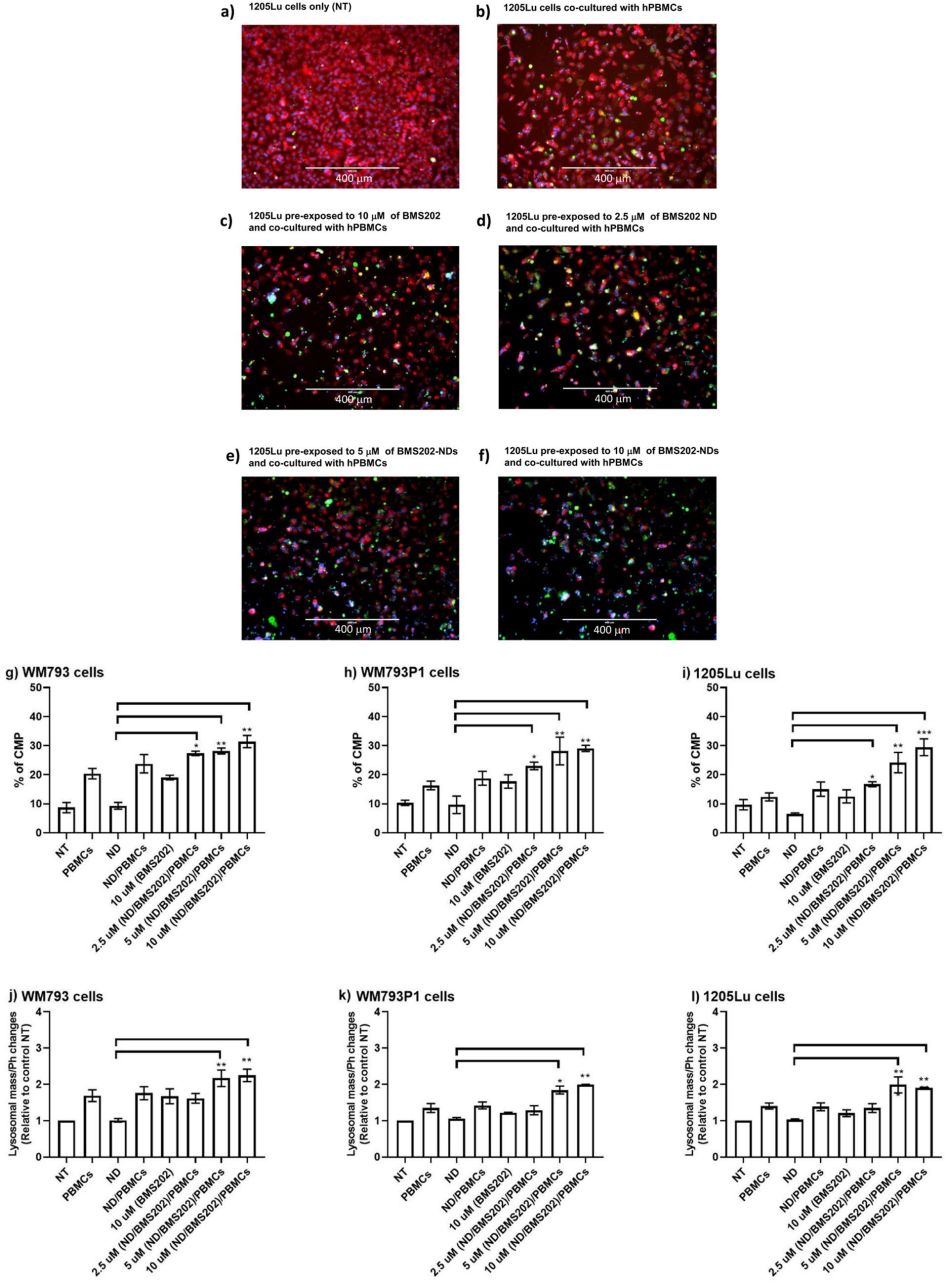
Cell Membrane Permeability (CMP) Damage and Lysosomal mass/pH changes. Melanoma cell lines were either not treated (NT) or treated with 2.5 µM, 5 µM, 10 µM of BMS202-NDs or to 10 µM of BMS202 alone for 6 h, then cells were co-cultured with hPBMCs for an additional 24 h. Cells were probed with CMP dye (green), Lysosomal mass/pH (red), and counterstained with Hoechst 33,342 (**a**–**f**). Cells were then scanned and analyzed using the Cytell™ imaging system and BioApp software. Changes in CMP (green) (**g**–**i**) and induction of lysosomal mass/pH changes (red) cells (**k**–**l**) were automatically counted, and data were presented as mean ± SEM (n = 3) and were analyzed using one‐way ANOVA coupled with a non-parametric Kruskal-Willis test, “*” for *p* < 0.05; “**” for *p* < 0.01; and “***” for *p* < 0.001.

Subsequently, we evaluated the toxic effects of hPBMCs on melanoma cells in the presence of nanocomplexes by means of cellular membrane permeability tests using the Cytell™ imaging system. A significant increase in the percentage of permeabilized cells was observed in all melanoma cell lines treated with nanocomplexes when they were co-cultured with hPBMCs (Fig. 4a–f). Similarly, this was in a concentration-dependent manner (2.5 µM nanocomplexes; *p* < 0.02, 5 µM nanocomplexes; *p* < 0.008, 10 µM nanocomplexes; *p* < 0.008) and cell type-dependent manner with the largest increase being in WM793 and WM793P1, the cell lines with low and medium metastatic potential respectively (Fig. 4g–l). Interestingly, we observed a statistically significant increase of cell permeabilization when WM793 cells were exposed to bare NDs and co-cultured with hPBMCs (*p* < 0.05). Melanoma cells with high metastatic (1205Lu) potential showed a statistically significant increase of cell permeabilization when exposed to hPBMCs with 2.5 µM nanocomplexes (*p* < 0.02), 5 µM nanocomplex (*p* < 0.001) and hPBMCs with 10 µM nanocomplex (*p* < 0.001). No changes were seen under treatment with either NDs alone or hPBMCs alone (Fig. 4g–l; Supplementary Table 3).

### Lysosomal mass and pH changes

The measurements of cell lysosomal mass and pH in response to various concentrations of nanocomplexes (BMS202-NDs) were performed by Cytell™ imaging tool. Changes of lysosomal mass and pH can designate an augmented rate of cytotoxicity (Fig. 4a–f). In this study, significant changes were distinguished in lysosomal mass/pH staining intensity following 6 h exposure to the medium (5 µM) and high (10 µM) concentrations of nanocomplexes that were co-cultured with hPBMCs. Minor changes of lysosomal mass and pH were observed in WM793 cells (low metastatic potential) WM793P1 (medium metastatic potential) and 1205Lu cell lines (high metastatic potential) under treatment with only hPBMCs and re-treated with NDs and co-cultured with hPBMCs (Fig. 4g–l; Supplementary Table 4).

### Detection of DNA damage post exposure to nanocomplexes and hPBMCs

**Figure 5:**
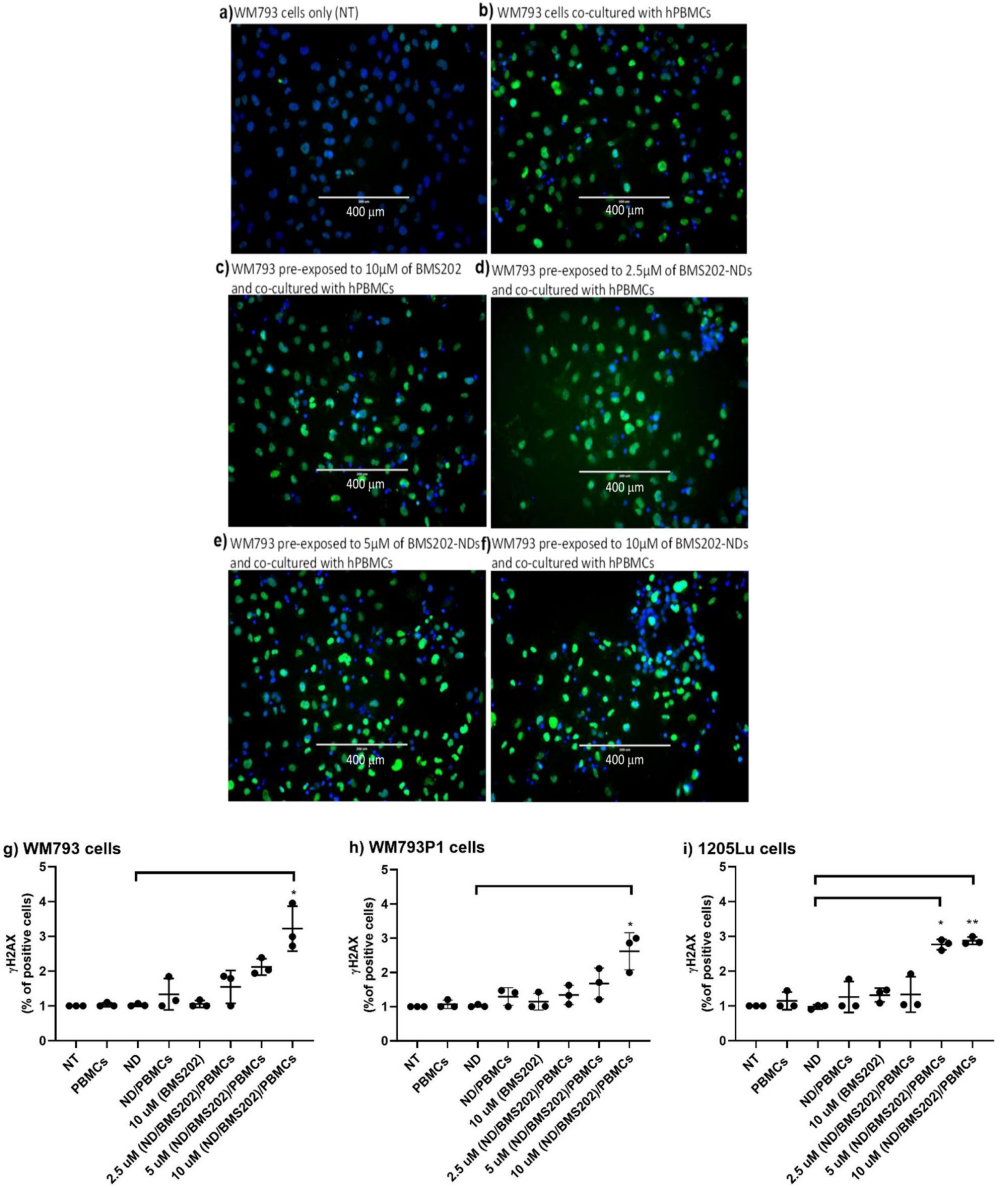
Detection of DNA damage post-exposure to ND/BMS202/PBMCs. Melanoma cell lines were either not treated (NT) or treated with 2.5 µM, 5 µM, 10 µM of BMS202-NDs or to/10 µM of BMS202 alone for 6 h, then cells were co-cultured with hPBMCs for an additional 24 h. Then cells were stained with anti- γ‑H2AX (green) and counterstained with Hoechst 33,342 (**a**–**f**). Then the expression of DNA damage marker (γ‑H2AX) was examined using the Cytell ™ imaging system, the number of γ‑H2AX-positive nuclei was counted (**g**–**i**) using BioApp software and data were presented as mean ± SEM (n = 3) and were analyzed using one‐way ANOVA coupled with a non-parametric Kruskal–Wallis test, “*” for *p* < 0.05 and “**” for *p* < 0.01.

**Figure 6:**
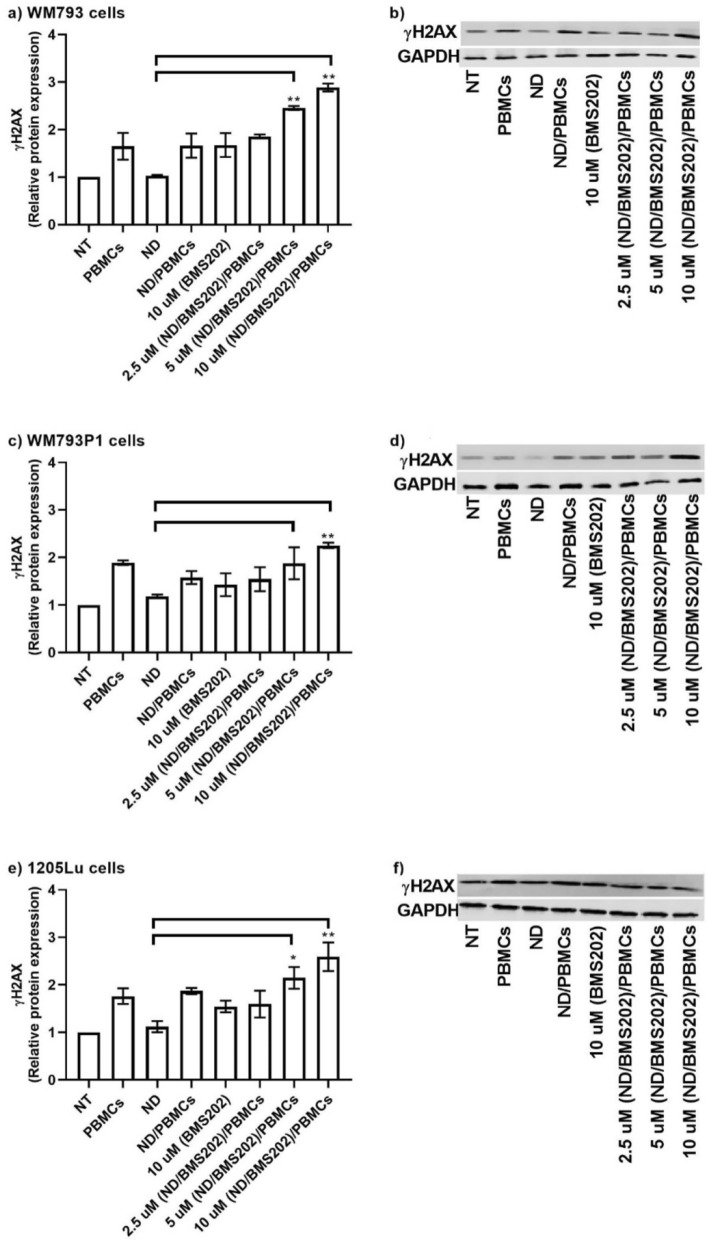
Upregulation of γ‑H2AX expression post exposure to ND/BMS202/PBMCs. Melanoma cell lines were either not treated (NT) or treated with 2.5 µM, 5 µM, 10 µM of BMS202-NDs or/to 10 µM of BMS202 alone for 6 h, then cells were co-cultured with hPBMCs for an additional 24 h. Cell lysates were harvested and then (40 μg) were resolved by SDS-PAGE and probed with anti- γ‑H2AX. Relative densitometric analysis of the individual bands was performed. The top band represents γH2aX and the bottom band represents GAPDH. Data were presented as mean ± SEM (n = 3) and were analyzed using one‐way ANOVA coupled with a non-parametric Kruskal–Wallis test carried out on the experimental data, with respect to the corresponding untreated controls (NT), “*” for *p* < 0.05, and “**” for *p* < 0.01.

Further, we examined the DNA damage upon exposure to our developed nanocomplex via the cellular expression of γ-H2AX. Briefly, melanoma cells were seeded in 96 wells/plate or T-25 flasks and were then exposed to nanocomplexes for 6 h and then co-cultured with hPBMCs as described above. Microscopic images (Fig. 5a–f) showed an increase in γ-H2AX expression in all melanoma cells after they were treated with nanocomplexes and co-cultured with hPBMCs, indicating increased DNA damage (Fig. 5g–i). This was seen at the medium and highest concentrations used. Only a minimal increase of γ-H2AX expression was induced by either hPBMCs alone or hPBMCs/NDs (Supplementary Table 5). Importantly, values for γ-H2AX expression measured 3 times by the immunoblotting methods demonstrate similar results to those observed by immunofluorescent staining in the various treatment conditions, providing further support that immunoblotting yields useful information regarding DNA damage levels (Fig. 6a–f; Supplementary Figs. S4 and S5). On the other hand, the melanoma cells treated with hPBMCs and/or pre-treated with ND and co-cultured with hPBMCs exhibited little, if any, difference in fluorescent staining or immunoblotting bands of γ-H2AX (Supplementary Table 6).

### Cleaved caspase 3 expression

**Figure 7:**
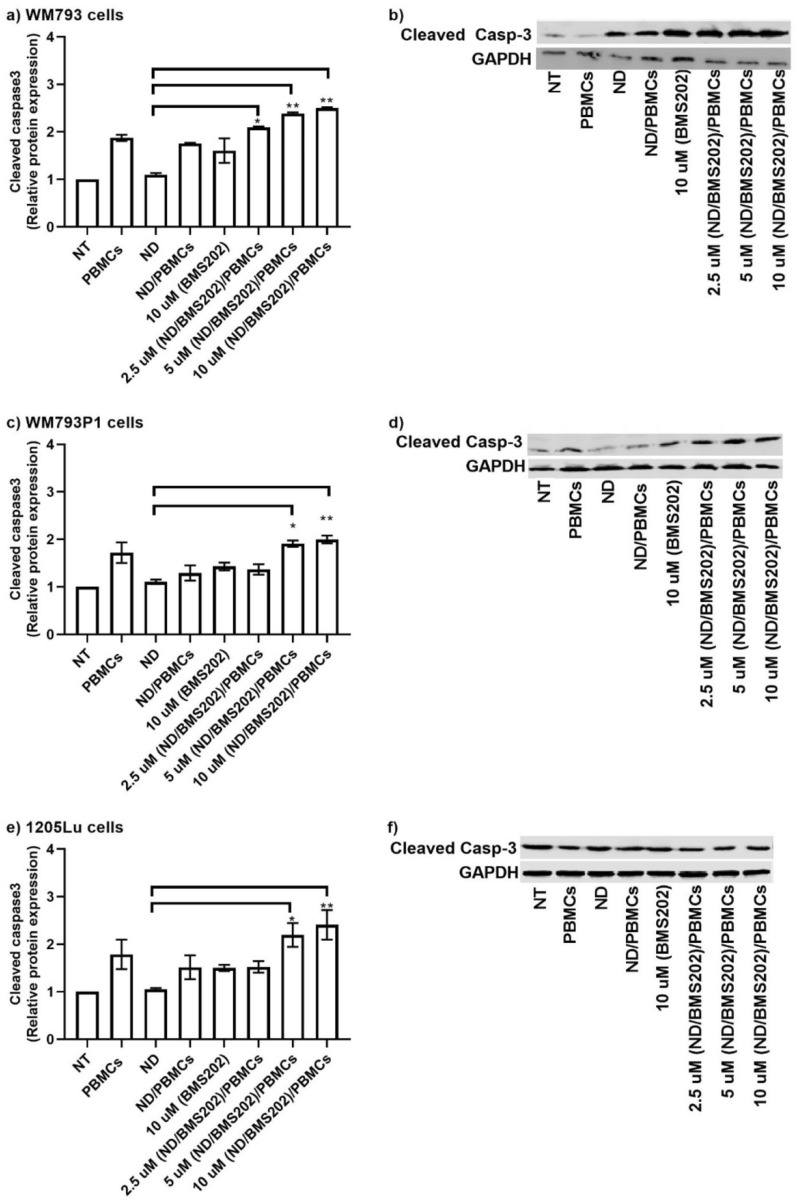
Cleaved caspase 3 expression. Melanoma cell lines were either not treated (NT) or treated with 2.5 µM, 5 µM, 10 µM of BMS202-NDs or/to 10 µM of BMS202 alone for 6 h, then cells were co-cultured with hPBMCs for an additional 24 h. Cell lysates were harvested and then (40 μg) were resolved by SDS-PAGE and probed with anti-cleaved caspase 3. Relative densitometric analysis of the individual bands was performed. The top row represents cleaved caspase 3 and the bottom row represents GAPDH. Data were presented as mean ± SEM (n = 3) and were analyzed using one‐way ANOVA coupled with a non-parametric Kruskal–Wallis test carried out on the experimental data, with respect to the corresponding untreated controls (NT), “*” for *p* < 0.05, and “**” for *p* < 0.01.

To evaluate if cells exposed to the nanocomplexes showed an apoptotic response, cleaved caspase 3 protein expression was quantified by measuring relative intensities using Odyssey software. We found that cleaved caspase 3 expression was significantly increased in nanocomplex-treated melanoma cells that were co-cultured with hPBMCs (Fig. 7a–f; Supplementary Fig. S6). The level of its expression was in a concentration-dependent manner. Interestingly, a significant increase of cleaved caspase 3 expression was also observed in WM793 and WM793P1 cells co-cultured with hPBMCs (Fig. 7a–f; Supplementary Fig. S6 and Supplementary Table 7).

## Discussion

Avoiding immune destruction is one of the hallmarks of cancer^51^. In the tumor microenvironment, cancer cells express PD-L1 which binds to the PD-1 receptor expressed on cells of the immune system, particularly T cells. Activation of the receptor leads to inhibition of the immune cell’s activity, which enables the cancer cells to evade immune-mediated destruction^52^. Therefore, the blockade of the PD-1/PD-L1 interaction reinvigorates the antitumor activity of these immune cells^12^. Monoclonal antibodies targeting PD-1/PD-L1 are widely used to treat a broad range of malignancies by activating T cell immunity^53^. However, only a proportion of patients respond to this treatment strategy^18,54,55^. This lack of efficacy may be associated with an increase of PD-L1 expression on cancer cells, elevated numbers of tumor-infiltrating immune cells in the tumor microenvironment, cancer cells with microsatellite instability or a mismatch-repair deficiency, cancers with an increased mutational burden and/or the existence of neoantigens^56^.

A common problem associated with the treatment of solid tumors centers on inadequate delivery of therapeutic small molecule inhibitors to the tumors^57^, and to this end, a large research effort is currently focused on the development of nanoparticle based approaches to enhance both delivery and drug efficacy^32,57^. A critical limitation concerning the clinical development of BMS-202 has been that due to its hydrophobicity and aggregation properties in aqueous media it may limited its overall efficacy as a checkpoint inhibitor^44^. As such, development of nanocarrier complexes that can both stabilize and enhance sustained delivery of this small molecule checkpoint inhibitor is of paramount importance and an area of active ongoing research^34,58–61^.

In this proof-of-concept study, we have used an in vitro cell line culture system coupled with hPBMCs to assess the treatment applicability of diamond-based nanomaterials (NDs) incorporated with immune checkpoint inhibitor for the treatment of melanoma. We developed NDs loaded with PD-1/PD-L1 inhibitor (BMS202) to target melanoma cells, enabling the blockade of the interaction between PD-L1 and its receptor and the subsequent enhancement of the immune response leading to destruction of the melanoma cells. To the best of our knowledge, this study is the first to investigate the immunotherapeutic benefits of loading NDs with a PD-1/PD-L1 small molecule inhibitor in a melanoma experimental setting.

NDs-based nanomaterials are promising material for nanocomplex formulations due to their attractive properties such as negligible cytotoxicity, great biocompatibility, high surface-to-volume ratio, facile functionalization, and low production cost. More precisely, their tendency to accumulate in tumor cells is beneficial for the treatment of cancer^38,39^. In drug delivery systems, nanomaterials are often coated with organic functionalities to enrich additional characteristics. In the past few years, Polyethylene glycol amine (PEG-NH2) is widely used for surface modification of nanomaterials, as it conveys hydrophilicity, provides stability against agglomeration, improves biocompatibility, decreases toxicity, reduces aggregation, and offers rapid absorption by the reticuloendothelial system with prolonged circulation time^38–43^. In this study we developed BMS202-functionalized nanocomplexes that had higher zeta potentials than the bare ND. Also compared to the bare NDs, we reported a decrease in the PDI of the ND-PEG-BMS202, which indicated that their dispersity was improved. Our results suggest that this strategy has some merit, but the mechanistic elements underpinning this remain to be elucidated.

Previously, it has been established that the melanoma cell line used WM-793 (and its clonal derivatives) express PD-L1^62^ at low levels. In this regard, it must be noted that the original FDA approval for the treatment of melanoma required a PD-L1 positivity of ≥ 1% using the PD-L1 pharmDx assay (although this has since been withdrawn as a requirement for treatment)^63^, suggesting that our melanoma cells could respond to BMS-202. Likewise, it could also be argued that the cytotoxic T-cells in our hPBMCs did not express PD-1. This seems unlikely as an analysis of PBMC subsets in both normal individuals and cancer patients found PD-1 expression across all hPBMCs (including CD8+ T Cells)^64^. As such we believe that our experimental system is technically functional. Future studies are required to determine which hPBMC subsets are responding in our experiments. hPBMC contain cytotoxic T cells and NK cells, which are likely to mediate cytotoxicity against tumor cells, as well as B cells, monocytes and small numbers of innate lymphoid cells which do not exhibit cytotoxicity.

It could be argued that Myeloid-derived suppressor cells (MDSCs) may also be contributing to the observed effects of our ND complex, and indeed the expansion of a subset of MDSC with immunosuppressive functions often occurs in cancer^65^. However, in this regard, the proportion of MDSCs found in peripheral blood from normal individuals has been estimated at 0.5%^65^, and whilst MDSCs may have some effect on our model as described, we believe that the vast majority of the effects observed can be attributed to CD8+ T cells. Nevertheless, future experiments will be required to investigate the potential role of MDSCs in our experimental model.

Conceptually, the time-frame of the responses observed suggest that they are mediated by memory T cells as the time frame is not sufficient for naïve T cell activation, and indeed a nanoparticle based study using immune therapy drugs (including BMS-202) backpacked onto adoptive T cell therapy in melanoma found that effector memory CD8+ T cells mediated anti-tumor immunity^37^. One limitation of our study is that it used unstimulated hPBMCs to assess tumor-immune responses. However, it must be noted that in a similar approach Russomanno et al., observed a similar small, enhanced reduction in cell viability for lung cancer cells when using unstimulated hPBMCs and BMS-202^26^. However, the responses that we observe for hPBMC may also relate in part to the cytotoxic activity of BMS-202. In this regard, pro-inflammatory cytokines such as interferon gamma (IFNγ) or other signals may be being released by the melanoma cells which may function to activate memory T cell responses.

We demonstrated a concentration-dependent increase in cell killing effect of hPBMCs on melanoma cells if pre-exposed to BMS202-ND. This was done by measuring several biological markers including cell viability changes, cell membrane permeability damage, lysosomal mass/pH alteration, γH2AX (a marker of DNA damage) and cleaved caspase 3 (a downstream apoptotic marker).

One of the common issues with melanoma research, particularly with regard to immune checkpoint inhibitor studies concerns the complexity of melanoma, and it could be argued that two-dimensional melanoma cell culture models such as the one used here are not suitable to answer the more complex scientific questions posed with respect to immune responses^66^. In addition, it could be argued that the genetic profiles of the cell lines (from the least aggressive WM793 to the most aggressive 1205Lu which may be the most resistant to treatment) may affect the responses observed, with potentially less obvious responses at the level of gamma-H2AX or Caspase-3 (Figs. 6 and 7). In this regard 1205Lu may have a more active DNA damage response pathway than the other cells and as such the level of gamma-H2AX is being repaired faster in these cells which reflects the western blot data shown in Fig. 6 and may have implications for any ICI based therapy. Moving forwards, it may therefore be necessary to conduct more functional studies on the DNA Damage Response (DDR) and Mismatch Excision Repair (MMR) pathways in these cells to determine if enhanced activity in any of these pathways is masking responses.

In addition, another particular limitation of our model is that the hPBMCs used are not HLA matched and traditional responses mediated by CD8 cells are antigen-specific and HLA-restricted. However, we believe that it represents a unique starting point to explore non-classical responses to ICI^67^. Indeed, initial studies of ICI focused on the positive role of CD8+ T-cell toxicity, but recent evidence suggests that the outcome of ICI (and in particular anti-PD-1/PD-L1 therapy) may actually also derive from non-CD8+ T cells (comprising both innate and adaptive immune cell types^68,69^.

Despite this, our results showed that most of the hPBMCs interacted with melanoma cell lines were CD8+ T cells. CD8^+^ T cells are the most prominent anti-tumor cells. Upon priming and activation by APCs, the CD8^+^ T cells differentiate into cytotoxic T lymphocytes (CTLs) and, through the exocytosis of perforin- and granzyme-containing granules, exert an efficient anti-tumoral attack, resulting in the direct destruction of target cells^70,71^. Support for targeting PD-L1 by ICI therapy in melanoma can be seen from an analysis of the TCGA-Melanoma datasets (TCGA-SKCM), where high expression of PD-L1 (CD274) is associated with better OS (Supplementary Fig. S7). In addition, an analysis of CD8+ T cells in the same dataset using TIMER2^72^ demonstrates that positive correlations were observed for all cell types examined (T cell CD8+ central memory cells; T cell CD8+ effector memory cells, T cell CD8+ naïve cells etc.) as shown in Supplementary Table 8.

However, since the hPBMC employed in the present investigation were not HLA-matched with the tumor cells, it is possible that the effector cells may be unconventional T cells, such as natural killer T cells, gamma-delta (γδ) T cells or mucosal-associated invariant T cells, all of which can express CD8, and, given the non-classical element of our in vitro model, as more recent analyses have shown that innate lymphocytes are novel targets of ICIs^68,73,74^. As such, it may be necessary to assess the effects of our ND-BMS202 complex on both natural killer (NK) cells and innate lymphoid cells (ILCs) following exposure. In the future, it may also be of benefit to first deplete the hPBMCs of CD8+ T cells prior to treatments to see if the results obtained are abrogated, as a way to show that CD8+ T cells are required for this effect, or to determine if NK or ILCs are central to the effects observed.

Alternatively, a key role for SOCS1 has been identified in regulating CD8+ T cell homeostasis, where it not only controls production of T cell stimulatory cytokines but also attenuates the sensitivity of CD8+ T cells to synergistic cytokine stimulation and critically antigen non-specific activation^75^, and may be a key regulator to examine in future studies. Alternatively, chronic exposure to IL-2 has recently been shown to induce CD8+ T cell exhaustion within tumor microenvironments^76^, and it may therefore be possible to potentially treat the hPBMCs to IL-2 to assess whether this may affect CD8+ T cell responses following exposure to ND-BMS202.

Our results demonstrated that blockade of PD-L1 in the presence of hPBMCs resulted in growth inhibition of melanoma cells to a greater extent than by hPBMCs alone. It has been previously reported that lysosome function is crucial for the growth and progression of varied human cancer types^77^, and pH-disrupting lysosomotropic agents such as hydroxychloroquine are effective anti-cancer agents in vitro and in vivo^78^. We, therefore, investigated if the co-culture of melanoma cells with hPBMCs following exposure to BMS202-ND indeed induced lysosomal mass/pH changes. Our results indicated that indeed there were obvious changes in lysosomal mass/pH. In fact, it has been reported that changes in lysosomal mass can be referred to as apoptotic responses, TNF-α, Fas and lysosomal photodamage^79–83^.

It has been we documented that NDs localized within the lysosomes post 24 h incubation time with no interfere with the cell viability^84,85^. Therefore, we may suggest that the changes of lysosomal mass reported in our study were depends on lysosomal co-localization of the BMS202-ND. In agreement with previous study, our unfunctionalized NDs did not inhibit the cell viability^84,85^.

We also showed that melanoma cells responded very well when we examined cell membrane damage indicating successful immune cell stimulation. It is known that the changes of cellular membrane permeability indicate the alterations of the physical condition of the cells^48^. It may also be that changes in the physicochemical properties of the nanodiamond complexes may be affecting cell membrane permeability.

Caspases play crucial roles in apoptosis and are intimately associated with cancer growth and prognosis^86^. Lack of activity and low expression levels of caspases make cancer cells resistant to microenvironmental stresses and therapies^87^. In this study, we examined the expression of cleaved caspase 3 and observed a significant increase of its expression in melanoma cells upon exposure to BMS202-conjugated NDs and hPBMCs. Blockade of PD-L1/PD-1 leads to the activation of T cell anti-tumor cytotoxicity and the production of IFNs that inhibit tumor cell growth and survival^68^. Therefore, we hypothesize that our treatment of melanoma cells with functionalized BMS202-conjugated NDs in the presence of hPBMCs led to the activation of hPBMCs, IFN-γ secretion and upregulation of cleaved caspase 3. While we have examined activation of caspase-3, in such a setting it may also be of benefit to measure levels of secreted IFN-γ to functionally validate this. Moreover, activation of T Cell mediated responses is often accompanied by the secretion/production of various cytokines^88^. Moving forwards, as we move to more complex 2D and 3D cancer models, we intend to assess a panel of basic cytokines to more effectively monitor T-Cell mediated responses.

Several treatments, such as chemotherapy and targeted therapies, need the whole IFN signal transduction pathway in cancer cells to exert their anti-tumor effects^89^. We observe an increase of γ-H2AX expression in melanoma cells following inhibition of PD-1/PD-L1 binding and immune cell-cancer cell interactions, indicating that DNA damage has been induced. It is well documented that monitoring DNA double strand breaks using γ-H2AX can be a sensitive indicator of drug efficiency^90,91^. γ-H2AX has been used experimentally in vitro^92^ and in vivo to measure drug toxicity, pharmacokinetics, and efficacy^93^. In agreement with previous studies, we reported a positive nuclear focus formation of γH2AX, which indicates double-stranded DNA damage repair induced by our developed nanocarrier complex comprising NDs/PD-L1 inhibitor.

We recognize the limitations of the current study in that it does not reflect the actual solid tumor microenvironment in solid tumors, such as melanoma. In this regard, better models with respect to three-dimensional in vitro approaches such as spheroids^66^ or patient derived organoids^66^ and in vivo animal studies which would probably require the use of murine melanoma syngeneic model^66^. Indeed, another possibility that has not been determined in the present study is whether or not pegylation used in the nanocarrier complex formation may in fact be stabilizing BMS202. Given that pegylation is regularly by the pharmaceutical industry to enhance drug stability^94^, it will be necessary also test this possibility by generating nanocarriers with different formulations. Another possibility could be that the ND complexes affect the pharmacokinetic stability of BMS-202. Early studies in mice monitored BMS-202 levels and found that BMS-202 is relatively stable with concentrations decreasing slowly in plasma and tumor^28^, but it may be that when complexed to nanodiamonds the stability of BMS-202 is increased allowing for longer target inhibition, and potentially a higher concentration of small molecule inhibitor closer to PD-L1 receptors as speculated by Zhang et al.^44^, and as such the zeta potential of the BMS202-NDs should be assessed. Other possibilities may be that as BMS202 has been recently been found to suppress collagen synthesis, α-SMA and collagen I expression in human fibroblasts^95^, it may also have such effects on collagen synthesis in cancer associated fibroblasts affecting the tumor microenvironment.

A potential limitation of the current ND-based platform is that it may require transfusion in patients via the blood stream. This may affect ND stability, and moving forwards it may be necessary to develop ND complexes based on our proof-of-concept complexes with increased stability and/or enhanced deliverability (via selective targeting). Alternatively, it may be possible to bypass this by developing a controlled release ointment for use on melanoma lesions similar to those previously described^96^.

Our results for this proof-of-concept nanodiamond delivery system show an improved anti-cancer immune response the mechanisms of which are still not fully understood. Clearly the next stage in their development will require better in vitro models such as the reconstructed human melanoma-in-skin models (Mel-RhS)^66,97^ utilizing patient cells and HLA-matched PBMCs, or in appropriate in vivo models to truly assess their potential as a novel approach to enhancing immune checkpoint-based therapy in melanoma.

## Conclusions

Our strategy was to generate an *in-vitro* non-cost-effective protocol comprising a small molecule PD-1/PD-L1 inhibitor-conjugated NDs and hPBMCs to advance the treatment of melanoma. Using this approach, we have demonstrated a significant immune response by inducing anti-tumor activity. We found that BMS202-conjugated NDs could be used as enhancement agents to reinforce the cytotoxic effect of the hPBMCs/CD8^+^ T cells. All key biological indicators of cellular functions used in this study approved our concept. Innovative future work that generates primary cultures of melanoma tumor cell lines and lines of specific cytotoxic T cells (such as iNKT cells) from hPBMCs might improve current cancer treatment strategies.

## Supplementary Information

Supplementary Information.

## Data Availability

All data generated or analyzed during this study are included in this published article [and its supplementary information files].
