# Supplementary material for: Proof of concept nanotechnological approach to in vitro targeting of malignant melanoma for enhanced immune checkpoint inhibition
Source: Sci Rep. 2023 May 8;13:7462. doi: 10.1038/s41598-023-34638-2 (PMC10167246; doi:10.1038/s41598-023-34638-2)
Supplement: Supplementary file 1 — Supplementary Information. [file 41598_2023_34638_MOESM1_ESM.pdf]

Supplementary Material

Proof of Concept Nanotechnological Approach to *in vitro* Targeting of Malignant Melanoma for Enhanced Immune Checkpoint Inhibition

Bandar Alharbi [1], Husam Qanash [1,*], Naif K. Binsaleh [1], Salem Alharthi [2], Abdulbaset Elasbali [3], Chandranil H. Gharekhan [4], Muhammad Mahmoud [5], Emmanouil Lioudakis [6], John J. O'Leary [7,8,9], Derek G. Doherty [8,10], Bashir M. Mohamed [8,9,10,*,†] and Steven G. Gray [8,11,†]

[1] Department of Medical Laboratory Science, College of Applied Medical Sciences, University of Ha'il, Hail 55476, Saudi Arabia

[2] Department of Biological Science, College of Arts and Science, Najran University, Najran 55461, Saudi Arabia

[3] Clinical Laboratory Science, College of Applied Medical Sciences-Qurayyat, Jouf University, Sakaka 42421, Saudi Arabia

[4] Amrita Center for Nanosciences and Molecular Medicine, Amrita Vishwa Vidyapeetham, India

[5] School of Medicine, Trinity College Dublin, Dublin, Ireland

[6] Department of Pharmacology and Therapeutics, School of Medicine, Trinity College Dublin, Dublin, Ireland

[7] Department of Histopathology, Trinity College Dublin, Emer Casey Molecular Pathology Research Laboratory, Coombe Women & Infants University Hospital, Dublin, Ireland

[8] Trinity St James's Cancer Institute, Dublin, Ireland

[9] Department of Obstetrics and Gynaecology, Trinity College Dublin, Dublin, Ireland

[10] Department of Immunology, Trinity College Dublin, Dublin, Ireland

[11] Department of Clinical Medicine, Trinity College Dublin, Dublin, Ireland

"†" Contributed equally to the direction of this study.

***** Correspondences:

Husam Qanash, Ph.D.
Department of Medical Laboratory Science
College of Applied Medical Sciences
University of Ha'il, Hail 55476, Saudi Arabia
E-mail: h.qanash@uoh.edu.sa

Bashir M. Mohamed, Ph.D.
Department of Immunology
Trinity College Dublin
Dublin, Ireland
Email: bashmohamed@gmail.com

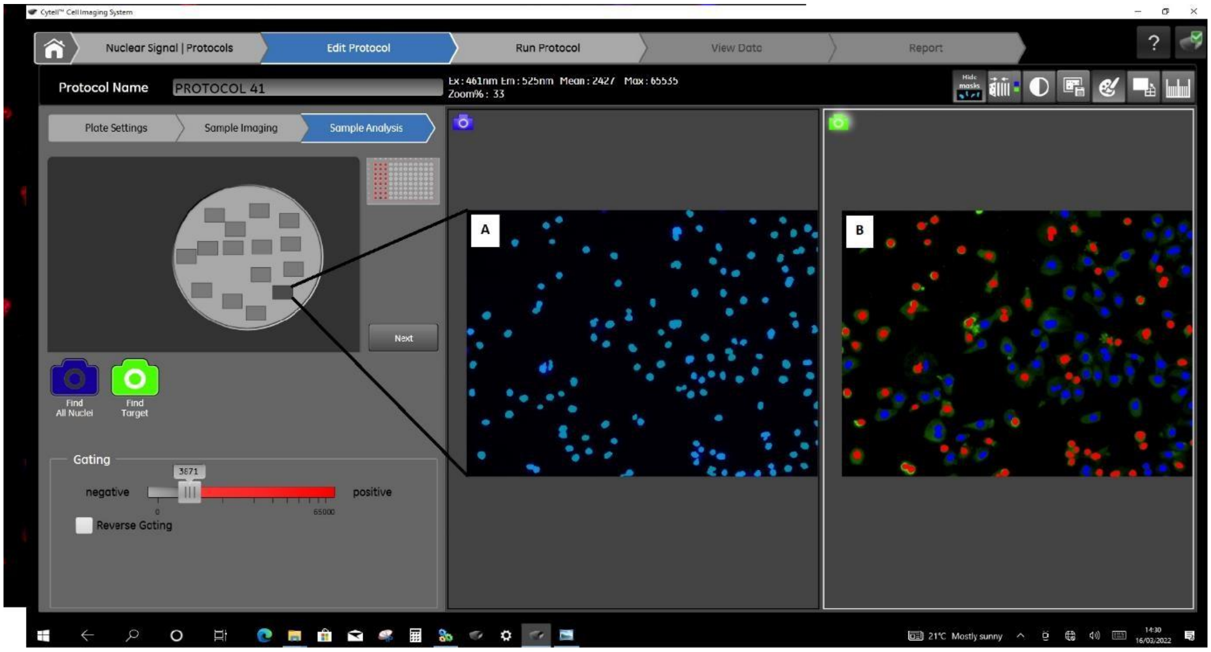

Supplementary Figure S1: Screen capture of an example of cell assessment using the Cytell Imaging System. Cells were scanned and analyzed utilizing the cell morphology bioApp (an algorithm-based software supplied by the company). Cells were either treated with BMS202-loaded NDs or BMS202 alone for 6h and then subsequently incubated with hPBMCs for an additional 24h. At this point cells were washed in PBS, fixed with 3% PFA and then probed with a cell membrane marker. After carrying out image calibration, fifteen random microscopic fields were sequentially acquired by the Cytell Imaging System at a magnification of 10×, maintaining a constant acquisition time, contrast, and brightness throughout the experiment. The bioApp algorithm automatically obtains the total number of cells, selects treatment-affected melanoma cells on the basis of morphological size and subsequently analyses them to determine: (a) the total number of stained nuclei (nuclei marked with light blue) and (b) the number of permeabilized (i.e., treatment affected melanoma cells) (selected cells masked with red) within the field.

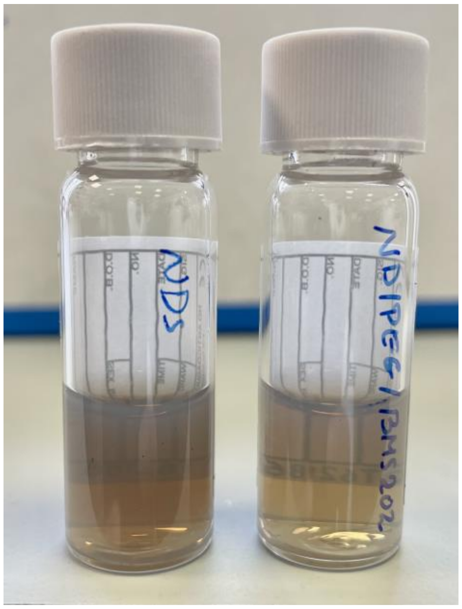

Supplementary Figure S2: Image of ND and ND-BMS202 taken 6 months' post functionalization, showing no aggregation or precipitation/colloid formation.

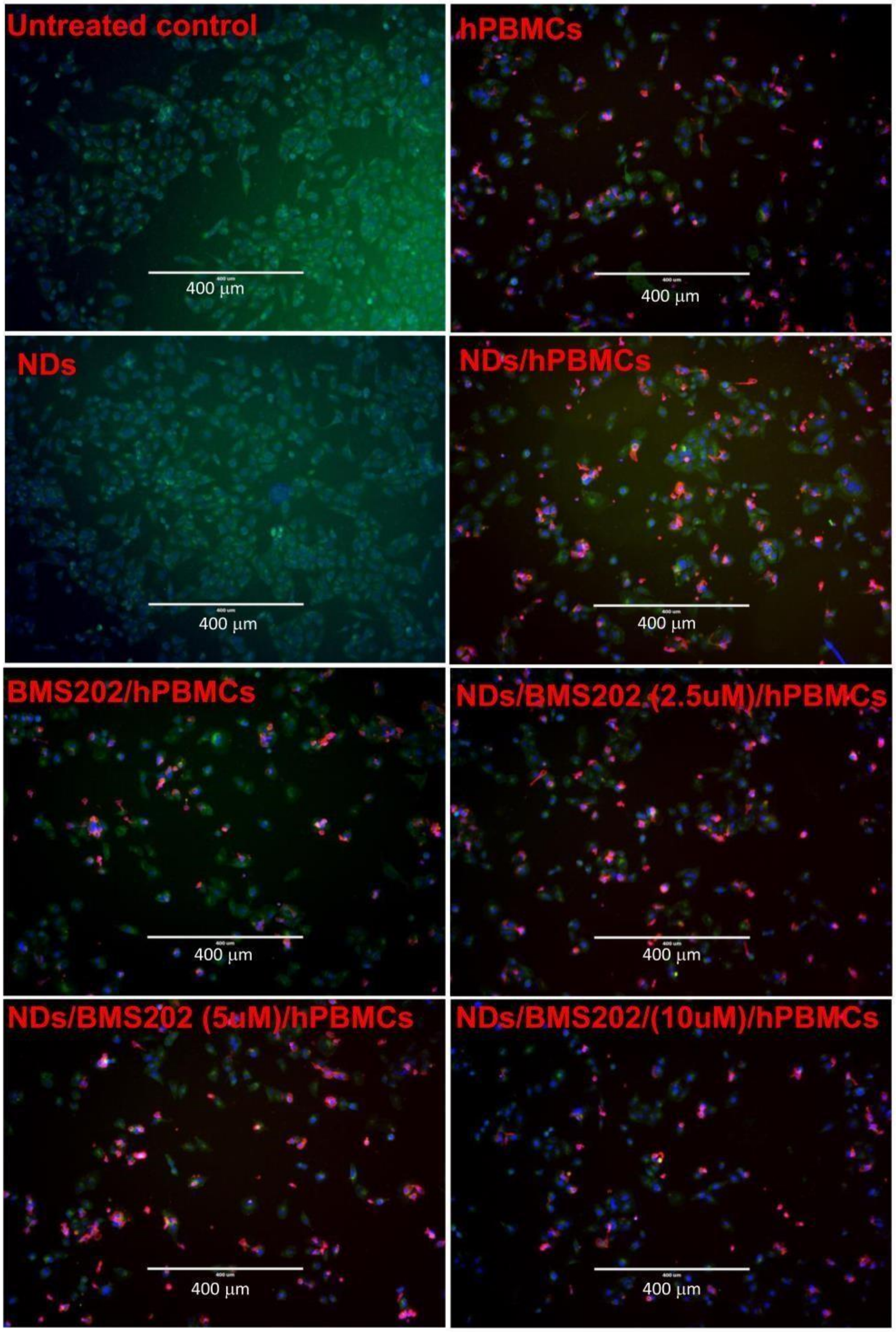

Supplementary Figure S3. hPBMCs/CD+ T cells and Melanoma cells interactions. Melanoma cells were exposed to BMS202-loaded NDs or BMS202 alone for 6h and then incubated with hPBMCs for an additional 24h. Cells then were stained with anti-CD8 antibodies (red), Cell tracker (green). Cells were counterstained with Hoechst 33342, (blue) for visualization of cell nuclei. Imaging was performed using an inverted microscope (20X).

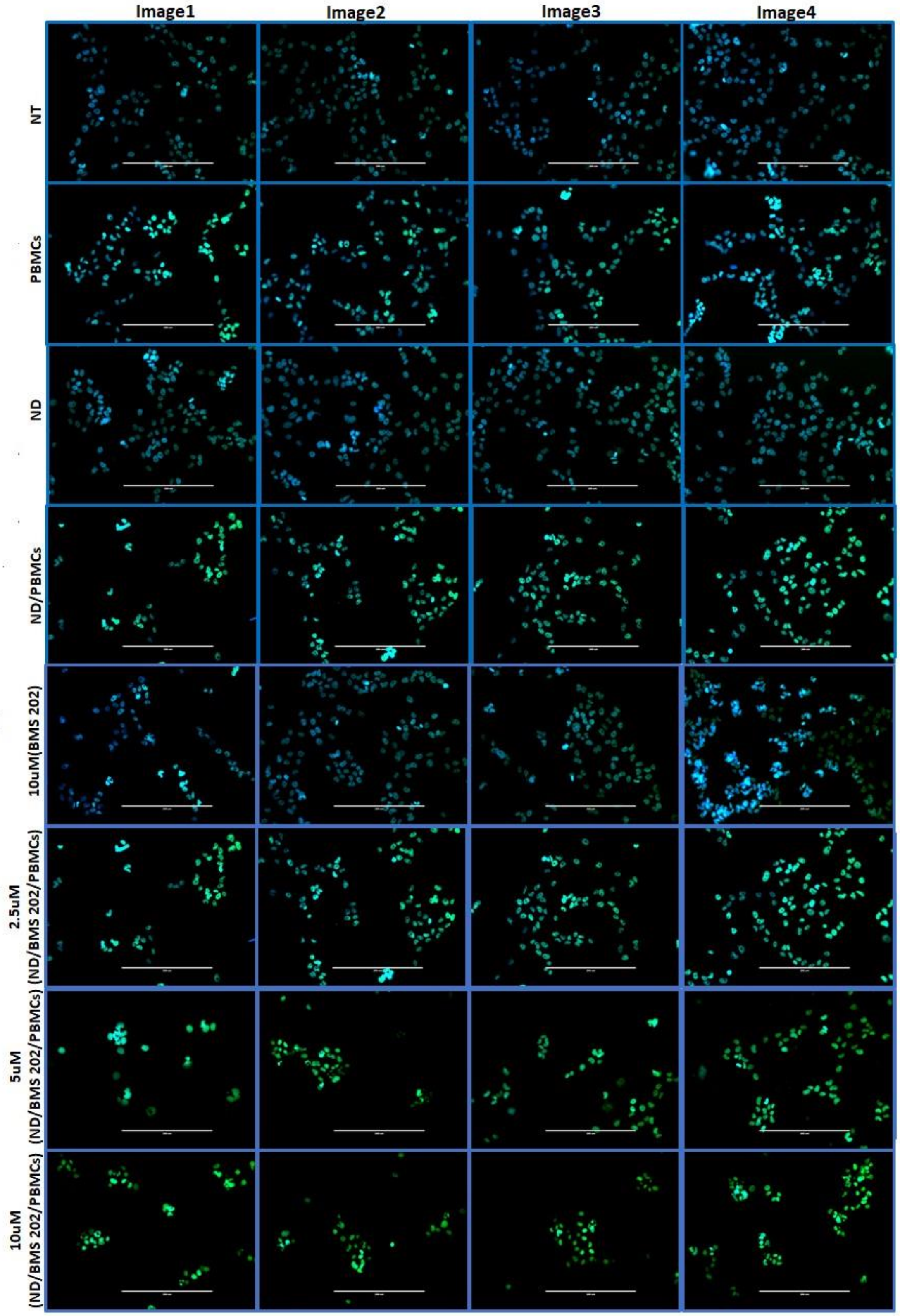

Supplementary Figure S4. An example of four different fields from each experiment looking at gamma H2AX (Blue- HOECHST; Green – gammaH2AX – scale bar represents 200 µm).

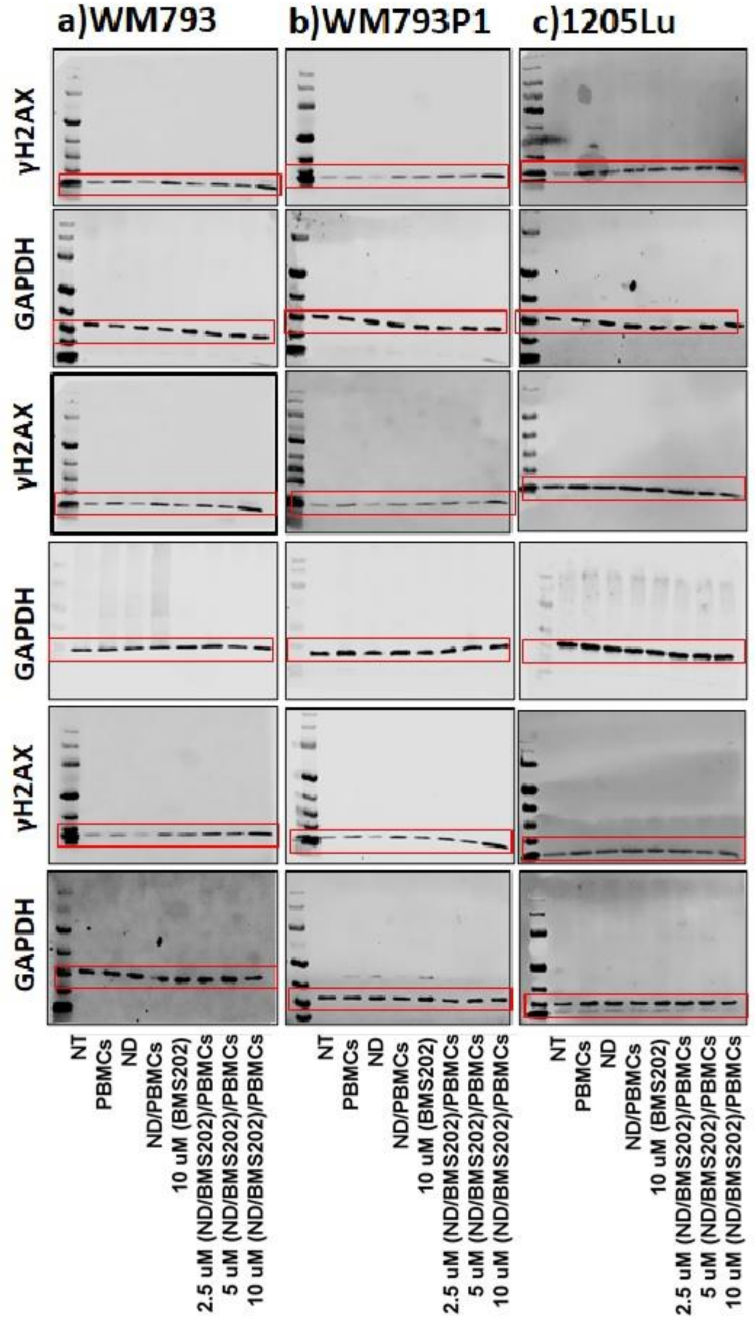

Supplementary Figure S5. Upregulation of γ-H2AX expression post exposure to ND/BMS202/PBMCs. Melanoma cell lines were either not treated (NT) or treated with 2.5μM, 5μM, 10μM of BMS202-loaded NDs or/to 10μM of BMS202 alone for 6 hours; then, cells were co-cultured with hPBMCs for an additional 24hours. Cell lysates were harvested and then (40μg) were resolved by SDS-PAGE and probed with anti- γ-H2AX. All blots were re-probed for GAPDH for normalization in downstream analyses, and a representative for one such is provided.

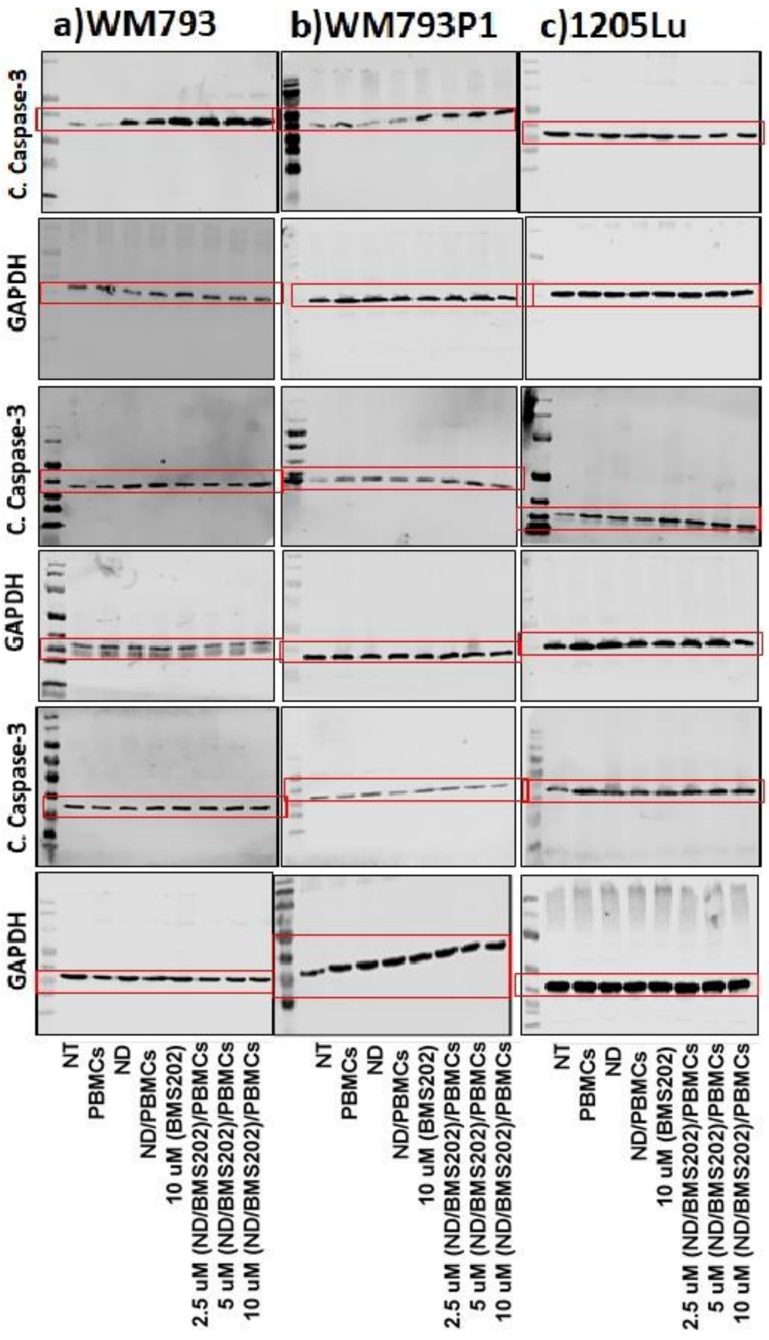

Supplementary Figure S6. Upregulation of cleaved caspase-3 expression post exposure to ND/BMS202/PBMCs. Melanoma cell lines were either not treated (NT) or treated with 2.5µM, 5µM, 10µM of BMS202-loaded NDs or/to 10µM of BMS202 alone for 6 hours; then, cells were co-cultured with hPBMCs for an additional 24hours. Cell lysates were harvested and then (40µg) were resolved by SDS-PAGE and probed with anti-cleaved caspase-3. All blots were re-probed for GAPDH for normalization in downstream analyses, and a representative for one such is provided.

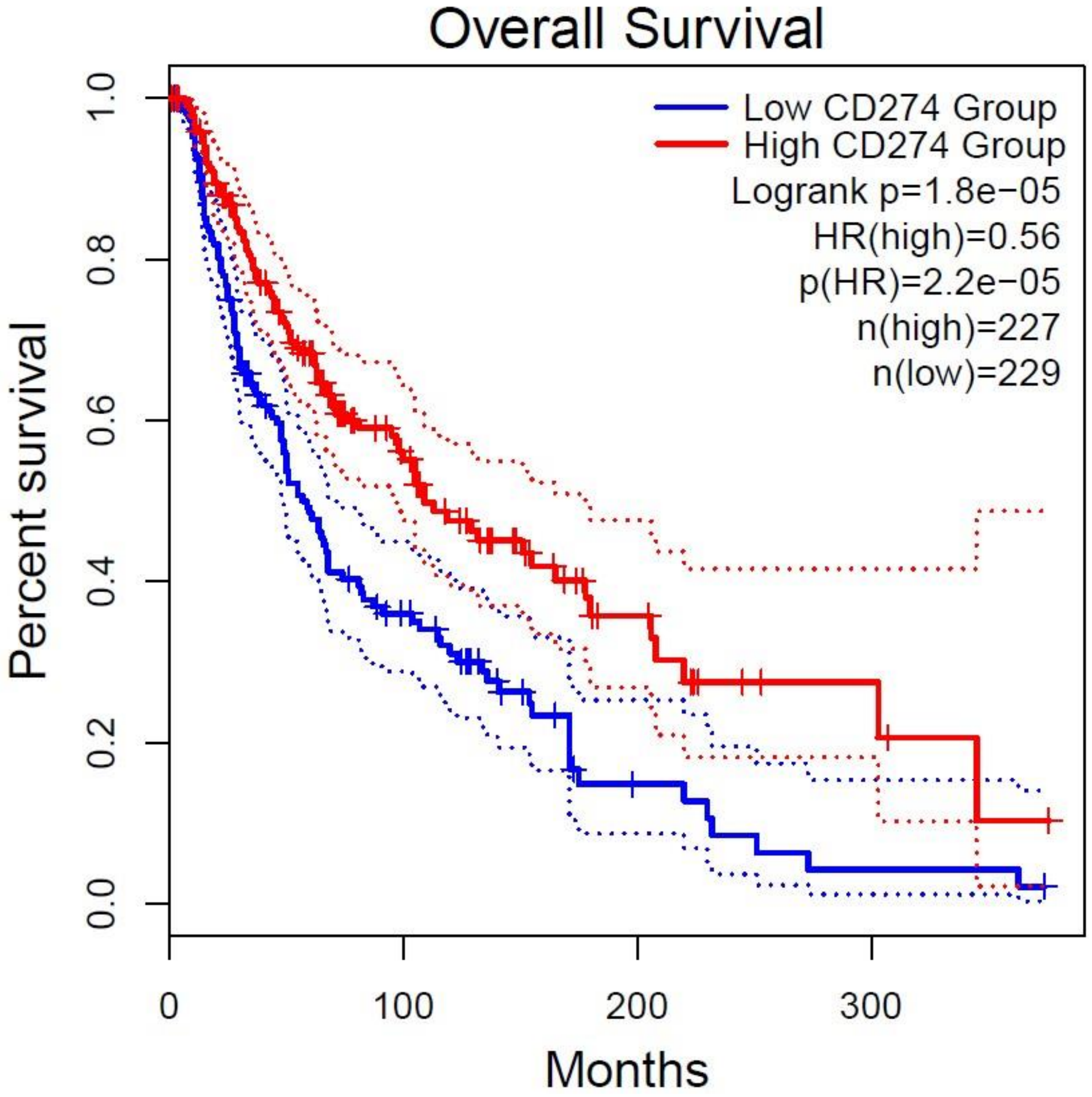

Supplementary Figure S7. Gepia 2 [1] analysis of the TCGA-SKCM dataset demonstrating that high expression of PD-L1 is associated with better OS.

Supplementary Table 1. Cell counts post-exposure to 2.5µM, 5µM, 10µM of BMS202-loaded NDs or to 10µM of BMS202 alone for 6h, then cells were co-cultured with hPBMCs for an additional 24h. Data analyzed using one-way ANOVA with Tukey's post-hoc test.

**Cell Count analysis**			
**WM793 cells**	**Treatment Conditions**	**Mean rank diff.**	**P Value**
	ND vs. NT	8.023	0.8
	ND vs. PBMCs	-44.97	<.001
	ND vs. ND/PBMCs	-1.773	<.001
	ND vs. 10 µM (BMS202)	-8.670	<.001
	**ND vs. 2.5 µM (ND/BMS202)/PBMCs**	12.01	<.001
	**ND vs. 5 µM (ND/BMS202)/PBMCs**	17.51	<.001
	**ND vs. 10 µM (ND/BMS202)/PBMCs**	20.27	<.001
**WM793P1 cells**			
	ND vs. NT	0.9	0.5
	ND vs. PBMCs	-30.43	<.001
	ND vs. ND/PBMCs	0.2767	<.001
	ND vs. 10 µM (BMS202)	-1.247	<.001
	ND vs. 2.5 µM (ND/BMS202)/PBMCs	18.68	<.001
	ND vs. 5 µM (ND/BMS202)/PBMCs	23.28	<.001
	ND vs. 10 µM (ND/BMS202)/PBMCs	34.24	<.001
**1205Lu cells**			
	ND vs. NT	5.9	0.9
	ND vs. PBMCs	-43.87	<.001
	ND vs. ND/PBMCs	-0.6400	<.001
	ND vs. 10 µM (BMS202)	-8.013	<.001
	ND vs. 2.5 µM (ND/BMS202)/PBMCs	-4.287	<.001
	ND vs. 5 µM (ND/BMS202)/PBMCs	8.740	<.001
	ND vs. 10 µM (ND/BMS202)/PBMCs	20.00	<.001

Supplementary Table 2. Cell counts post-exposure to 2.5µM, 5µM, 10µM of BMS202-loaded NDs or to 10µM of BMS202 alone for 6h, then cells were co-cultured with hPBMCs for an additional 24h. Data was analyzed using one-way ANOVA with Tukey's post-hoc test.

**Cell Count analysis**			
**WM793 cells**	**Treatment Conditions**	**Mean rank diff.**	**P Value**
	ND/PBMCs vs. 10 µM (BMS202)	-6.897	.995
	ND/PBMCs vs. 2.5 µM (ND/BMS202)/PBMCs	13.78	.29
	ND/PBMCs vs. 5 µM (ND/BMS202)/PBMCs	19.29	.056
	ND/PBMCs vs. 10 µM (ND/BMS202)/PBMCs	22.04	**.022**
**WM793P1 cells**			
	ND/PBMCs vs. 10 µM (BMS202)	-1.523	>.999
	ND/PBMCs vs. 2.5 µM (ND/BMS202)/PBMCs	18.40	**.018**
	ND/PBMCs vs. 5 µM (ND/BMS202)/PBMCs	23.00	**.003**
	ND/PBMCs vs. 10 µM (ND/BMS202)/PBMCs	33.96	**<.001**
**1205Lu cells**			
	ND/PBMCs vs. 10 µM (BMS202)	-7.373	.836
	ND/PBMCs vs. 2.5 µM (ND/BMS202)/PBMCs	-3.647	.996
	ND/PBMCs vs. 5 µM (ND/BMS202)/PBMCs	9.380	.625
	ND/PBMCs vs. 10 µM (ND/BMS202)/PBMCs	20.64	**.019**

Supplementary Table 3. Cell membrane permeabilization (CMP)changes post-exposure to 2.5µM, 5µM, 10µM of BMS202-loaded NDs or to 10µM of BMS202 alone for 6h, then cells were co-cultured with hPBMCs for an additional 24h. Data analyzed using one-way ANOVA coupled with a non-parametric Kruskal-Willis test.

**CMP analysis**			
**WM793 cells**	**Treatment conditions**	**Mean rank diff.**	**P Value**
	ND vs. NT	1	0.95
	ND vs. PBMCs	-7	0.23
	ND vs. ND/PBMCs	-11	0.07
	ND vs. 10 µM (BMS202)	-6	0.27
	**ND vs. 2.5 µM (ND/BMS202)/PBMCs**	**-12**	**0.02**
	**ND vs. 5 µM (ND/BMS202)/PBMCs**	**-14.67**	**0.008**
	**ND vs. 10 µM (ND/BMS202)/PBMCs**	**-18.33**	**0.002**
**WM793P1 cells**			
	ND vs. NT	0	>0.99
	ND vs. PBMCs	-5.667	0.33
	ND vs. ND/PBMCs	-8	0.17
	ND vs. 10 µM (BMS202)	-7	0.23
	**ND vs. 2.5 µM (ND/BMS202)/PBMCs**	**-13.33**	**0.02**
	**ND vs. 2.5 µM (ND/BMS202)/PBMCs**	**-16**	**0.006**
	**ND vs. 2.5 µM (ND/BMS202)/PBMCs**	**-18**	**0.002**
**1205Lu cells**			
	ND vs. NT	-4	0.49
	ND vs. PBMCs	-7.667	0.18
	ND vs. ND/PBMCs	-10.33	0.07
	ND vs. 10 µM (BMS202)	-8	0.17
	**ND vs. 2.5 µM (ND/BMS202)/PBMCs**	**-13**	**0.02**
	**ND vs. 5 µM (ND/BMS202)/PBMCs**	**-18.33**	**0.001**
	**ND vs. 10 µM (ND/BMS202)/PBMCs**	**-20**	**<0.001**

Supplementary Table 4. Changes in lysosomal mass/pH post-exposure to 2.5µM, 5µM, 10µM of BMS202-loaded NDs or to 10µM of BMS202 alone for 6h, then cells were co-cultured with hPBMCs for an additional 24h. Data analyzed using one-way ANOVA coupled with a non-parametric Kruskal-Willis test.

**Lysosomal mass/pH analysis**			
**WM793 cells**	**Treatment conditions**	**Mean rank diff.**	**P Value**
	ND vs. NT	1	0.86
	ND vs. PBMCs	-9	0.12
	ND vs. ND/PBMCs	-9.667	0.09
	ND vs. 10 µM (BMS202)	-9.667	0.09
	ND vs. 2.5 µM (ND/BMS202)/PBMCs	-7.667	0.18
	**ND vs. 5 µM (ND/BMS202)/PBMCs**	**-15.33**	**0.008**
	**ND vs. 10 µM (ND/BMS202)/PBMCs**	**-17.67**	**0.002**
**WM793P1 cells**			
	ND vs. NT	3.333	0.56
	ND vs. PBMCs	-7	0.22
	ND vs. ND/PBMCs	-9.667	0.09
	ND vs. 10 µM (BMS202)	-5	0.39
	ND vs. 2.5 µM (ND/BMS202)/PBMCs	-6.667	0.25
	**ND vs. 5 µM (ND/BMS202)/PBMCs**	**-14.67**	**0.01**
	**ND vs. 10 µM (ND/BMS202)/PBMCs**	**-17.67**	**0.002**
**1205Lu cells**			
	ND vs. NT	3.333	0.56
	ND vs. PBMCs	-8.667	0.13
	ND vs. ND/PBMCs	-8	0.17
	ND vs. 10 µM (BMS202)	-4.333	0.45
	ND vs. 2.5 µM (ND/BMS202)/PBMCs	-7.333	0.2
	**ND vs. 5 µM (ND/BMS202)/PBMCs**	**-16**	**0.006**
	**ND vs. 10 µM (ND/BMS202)/PBMCs**	**-16.33**	**0.005**

Supplementary Table 5. Immunohistochemical examination of DNA damage marker (γH2AX) post-exposure to 2.5µM, 5µM, 10µM of BMS202-loaded NDs or to 10µM of BMS202 alone for 6h, then cells were co-cultured with hPBMCs for an additional 24h. Data analyzed using one-way ANOVA coupled with a non-parametric Kruskal-Willis test.

**γH2AX expression**			
WM793 cells	**Treatment conditions**	**Mean rank diff.**	**P Value**
	ND vs. NT	6	0.29
	ND vs. PBMCs	3.333	0.55
	ND vs. ND/PBMCs	-1	0.86
	ND vs. 10 µM (BMS202)	2.667	0.64
	ND vs. 2.5 µM (ND/BMS202)/PBMCs	-2	0.72
	ND vs. 5 µM (ND/BMS202)/PBMCs	-9	0.11
	**ND vs. 10 µM (ND/BMS202)/PBMCs**	**-12**	**0.03**
**WM793P1 cells**			
	ND vs. NT	4.333	0.44
	ND vs. PBMCs	1.5	0.79
	ND vs. ND/PBMCs	-4	0.48
	ND vs. 10 µM (BMS202)	-1.333	0.81
	ND vs. 2.5 µM (ND/BMS202)/PBMCs	-6.167	0.28
	ND vs. 5 µM (ND/BMS202)/PBMCs	-9.833	0.08
	**ND vs. 10 µM (ND/BMS202)/PBMCs**	**-13.83**	**0.01**
**1205Lu cells**			
	ND vs. NT	1.667	0.77
	ND vs. PBMCs	-1.667	0.77
	ND vs. ND/PBMCs	-2.333	0.68
	ND vs. 10 µM (BMS202)	-7.667	0.18
	ND vs. 2.5 µM (ND/BMS202)/PBMCs	-7	0.22
	**ND vs. 5 µM (ND/BMS202)/PBMCs**	**-14**	**0.01**
	**ND vs. 10 µM (ND/BMS202)/PBMCs**	**-15.67**	**0.006**

Supplementary Table 6. Immunoblotting examination of DNA damage marker (γH2AX) post-exposure to 2.5µM, 5µM, 10µM of BMS202-loaded NDs or to 10µM of BMS202 alone for 6h, then cells were co-cultured with hPBMCs for an additional 24h. Data analyzed using one-way ANOVA coupled with a non-parametric Kruskal-Willis test.

**γH2AX expression**			
**WM793 cells**	**Treatment conditions**	**Mean rank diff.**	**P Value**
	ND vs. NT	3	0.6
	ND vs. PBMCs	-7	0.22
	ND vs. ND/PBMCs	-7.333	0.2
	ND vs. 10 µM (BMS202)	-8	0.17
	ND vs. 2.5 µM (ND/BMS202)/PBMCs	-7.667	0.18
	**ND vs. 5 µM (ND/BMS202)/PBMCs**	**-15**	**0.009**
	**ND vs. 10 µM (ND/BMS202)/PBMCs**	**-18**	**0.002**
**WM793P1 cells**			
	ND vs. NT	5.333	0.36
	ND vs. PBMCs	-9.333	0.11
	ND vs. ND/PBMCs	-5.333	0.36
	ND vs. 10 µM (BMS202)	-2.667	0.64
	ND vs. 2.5 µM (ND/BMS202)/PBMCs	-4.667	0.42
	ND vs. 5 µM (ND/BMS202)/PBMCs	-9.667	0.09
	**ND vs. 10 µM (ND/BMS202)/PBMCs**	**-15**	**0.009**
**1205Lu cells**			
	ND vs. NT	1.333	0.8
	ND vs. PBMCs	-10	0.08
	ND vs. ND/PBMCs	-11	0.06
	ND vs. 10 µM (BMS202)	-6	0.3

	ND vs. 2.5 µM (ND/BMS202)/PBMCs	-8	0.17
	**ND vs. 5 µM (ND/BMS202)/PBMCs**	**-14**	**0.02**
	**ND vs. 10 µM (ND/BMS202)/PBMCs**	**-17.67**	**0.002**

Supplementary Table 7. Cleaved Caspase-3 Immunoblotting examination of cleaved caspase-3 post-exposure to 2.5µM, 5µM, 10µM of BMS202-loaded NDs or to 10µM of BMS202 alone for 6h, then cells were co-cultured with hPBMCs for an additional 24h. Data analyzed using one-way ANOVA coupled with a non-parametric Kruskal-Willis test.

**Cleaved Caspase-3**			
**WM793 cells**	**Treatment conditions**	**Mean rank diff.**	**P Value**
	ND vs. NT	3.667	0.5
	ND vs. PBMCs	-7.333	0.2
	ND vs. ND/PBMCs	-4.333	0.45
	ND vs. 10 µM (BMS202)	-3.667	0.53
	**ND vs. 2.5 µM (ND/BMS202)/PBMCs**	**-11.33**	**0.05**
	**ND vs. 5 µM (ND/BMS202)/PBMCs**	**-14.33**	**0.01**
	**ND vs. 10 µM (ND/BMS202)/PBMCs**	**-17.33**	**0.003**
**WM793P1 cells**			
	ND vs. NT	4	0.49
	ND vs. PBMCs	-11	0.06
	ND vs. ND/PBMCs	-3.667	0.53
	ND vs. 10 µM (BMS202)	-6.333	0.27
	ND vs. 2.5 µM (ND/BMS202)/PBMCs	-5.667	0.33
	**ND vs. 5 µM (ND/BMS202)/PBMCs**	**-14.33**	**0.01**
	**ND vs. 10 µM (ND/BMS202)/PBMCs**	**-15**	**0.009**
**1205Lu cells**			
	ND vs. NT	3.667	0.5
	ND vs. PBMCs	-10.67	0.06
	ND vs. ND/PBMCs	-6.333	0.27
	ND vs. 10 µM (BMS202)	-5.333	0.36
	ND vs. 2.5 µM (ND/BMS202)/PBMCs	-6.667	0.25
	**ND vs. 5 µM (ND/BMS202)/PBMCs**	**-14.33**	**0.01**
	**ND vs. 10 µM (ND/BMS202)/PBMCs**	**-15**	**0.009**

Supplementary Table 8: TIMER2 analysis [2] of CD8+ T Cell infiltrations associated with PD-L1 expression in melanoma, using Purity Adjustment and partial Spearman's correlation association analysis.

Cancer*	infiltrates	rho	p	adj.p
SKCM (n=471)	T cell CD8+ central memory_XCELL	0.566358544	$4.01x10^{-40}$	**$2.29\ x10^{-38}$**
SKCM (n=471)	T cell CD8+ effector memory_XCELL	0.215061728	$3.50\ x10^{-06}$	**$9.85\ x10^{-06}$**
SKCM (n=471)	T cell CD8+ naive_XCELL	0.232315367	$5.12\ x10^{-07}$	**$1.64\ x10^{-06}$**
SKCM (n=471)	T cell CD8+_CIBERSORT	0.328076294	$6.28\ x10^{-13}$	**$3.69\ x10^{-12}$**
SKCM (n=471)	T cell CD8+_CIBERSORT-ABS	0.575992493	$9.58\ x10^{-42}$	**$6.39\ x10^{-40}$**
SKCM (n=471)	T cell CD8+_EPIC	0.482714665	$4.73\ x10^{-28}$	**$1.05\ x10^{-26}$**
SKCM (n=471)	T cell CD8+_MCPCOUNTER	0.589240881	$4.60\ x10^{-44}$	**$4.60\ x10^{-42}$**
SKCM (n=471)	T cell CD8+_QUANTISEQ	0.581735214	$9.76\ x10^{-43}$	**$7.80\ x10^{-41}$**
SKCM (n=471)	T cell CD8+_TIMER	0.435464133	$1.43\ x10^{-22}$	**$1.91\ x10^{-21}$**
SKCM (n=471)	T cell CD8+_XCELL	0.460423381	$2.33\ x10^{-25}$	**$4.05\ x10^{-24}$**
SKCM-Metastasis (n=368)	T cell CD8+ central memory_XCELL	0.564857591	$3.17\ x10^{-31}$	**$9.05\ x10^{-30}$**
SKCM-Metastasis (n=368)	T cell CD8+ effector memory_XCELL	0.216702775	$3.93\ x10^{-05}$	**$9.52\ x10^{-05}$**
SKCM-Metastasis (n=368)	T cell CD8+ naive_XCELL	0.267007176	$3.42\ x10^{-07}$	**$1.12\ x10^{-06}$**
SKCM-Metastasis (n=368)	T cell CD8+_CIBERSORT	0.361354854	$2.33\ x10^{-12}$	**$1.29\ x10^{-11}$**
SKCM-Metastasis (n=368)	T cell CD8+_CIBERSORT-ABS	0.586600888	$4.18\ x10^{-34}$	**$1.67\ x10^{-32}$**
SKCM-Metastasis (n=368)	T cell CD8+_EPIC	0.492610038	$4.86\ x10^{-23}$	**$6.71\ x10^{-22}$**
SKCM-Metastasis (n=368)	T cell CD8+_MCPCOUNTER	0.583466984	$1.12\ x10^{-33}$	**$4.08\ x10^{-32}$**
SKCM-Metastasis (n=368)	T cell CD8+_QUANTISEQ	0.590278124	$1.30\ x10^{-34}$	**$5.76\ x10^{-33}$**
SKCM-Metastasis (n=368)	T cell CD8+_TIMER	0.426302719	$4.60\ x10^{-17}$	**$3.83\ x10^{-16}$**

SKCM-Metastasis (n=368)	T cell CD8+_XCELL	0.460007367	6.14 $x10^{-20}$	**7.01 $x10^{-19}$**
SKCM-Primary (n=103)	T cell CD8+ central memory_XCELL	0.445177992	2.76 $x10^{-06}$	**7.87 $x10^{-06}$**
SKCM-Primary (n=103)	T cell CD8+ effector memory_XCELL	0.134113696	0.178990971	0.225856114
SKCM-Primary (n=103)	T cell CD8+ naive_XCELL	0.165247385	0.096958757	0.129710712
SKCM-Primary (n=103)	T cell CD8+_CIBERSORT	0.205550386	0.038213425	0.056403579
SKCM-Primary (n=103)	T cell CD8+_CIBERSORT-ABS	0.380202498	8.09 $x10^{-05}$	**0.000189317**
SKCM-Primary (n=103)	T cell CD8+_EPIC	0.41602783	1.37 $x10^{-05}$	**3.58 $x10^{-05}$**
SKCM-Primary (n=103)	T cell CD8+_MCPCOUNTER	0.452530063	1.80 $x10^{-06}$	**5.24 $x10^{-06}$**
SKCM-Primary (n=103)	T cell CD8+_QUANTISEQ	0.376889198	9.44 $x10^{-05}$	**0.000218329**
SKCM-Primary (n=103)	T cell CD8+_TIMER	0.40274524	2.71 $x10^{-05}$	**6.82 $x10^{-05}$**
SKCM-Primary (n=103)	T cell CD8+_XCELL	0.317475907	0.001148471	**0.00224092**

* - SKCM – Skin Cutaneous Melanoma

Tumor purity is a major confounding factor in TIMER2 analysis [2], since most immune cell types are negatively correlated with tumor purity. Therefore, it is recommended that users to select the “Purity Adjustment” option, which will use the partial Spearman’s correlation to perform this association analysis.

Supplementary References

1 Tang, Z., Kang, B., Li, C., Chen, T. & Zhang, Z. GEPIA2: an enhanced web server for large-scale expression profiling and interactive analysis. *Nucleic Acids Res* **47**, W556-w560, doi:10.1093/nar/gkz430 (2019).

2 Li, T. *et al.* TIMER2.0 for analysis of tumor-infiltrating immune cells. *Nucleic Acids Res* **48**, W509-w514, doi:10.1093/nar/gkaa407 (2020).
